# Applying neural networks as direct controllers in position and trajectory tracking algorithms for holonomic UAVs

**DOI:** 10.1038/s41598-025-97215-9

**Published:** 2025-04-12

**Authors:** Cezary Kownacki, Slawomir Romaniuk, Marcin Derlatka

**Affiliations:** 1 Department of Automation of Manufacturing Processes, Bialystok University of Technology, 15-351 Białystok, Poland; 2 Department of Automatic Control and Robotics, Bialystok University of Technology, 15-351 Białystok, Poland; 3 Institute of Biomedical Engineering, Bialystok University of Technology, 15-351 Białystok, Poland

**Keywords:** Artificial potential field, UAV, Quadcopter, Path tracking, Neural networks, Deep neural networks, Multilayer perceptron, Residual neural network, Aerospace engineering, Computer science, Electrical and electronic engineering

## Abstract

This study compares different neural networks as standalone control algorithms for position and trajectory tracking in holonomic UAVs, specifically quadcopters. The research’s novelty lies in applying these algorithms directly for control. A position-tracking algorithm based on the artificial potential field method generated extensive training and validation datasets, simulating the tracked point’s diverse trajectory shapes and velocities. The most popular neural network architectures were evaluated on the basis of their trajectory tracking accuracy and computational performance, i.e. single-layer regression networks and double-layer perceptron regression networks, deep neural networks, and residual networks. The results highlight that DNNs achieved the highest trajectory tracking accuracy, as measured by root mean squared errors (1.0830) and correlation coefficients (0.9624 given as Pearson’s correlation) while providing satisfactory results and stable flight across untrained scenarios, in opposite to other neural networks. However, simpler architectures, such as single-layer perceptrons, exhibit significantly lower latency, making them suitable for real-time applications despite slightly reduced accuracy. In contrast, ResNet architectures underperformed in terms of accuracy and latency, emphasizing the importance of selecting architectures on the basis of specific control objectives. This study demonstrates that deep neural networks can directly control quadcopters, eliminating the need for conventional control algorithms for UAV position-tracking applications, provided sufficient learning data is available. The proposed approach ensures accurate trajectory tracking, effectively handling sudden turns while maintaining stable flight. These findings highlight the potential of neural networks for UAV control, balancing computational efficiency with high precision and reliability.

## Introduction

Currently , greater focus is placed on the research and development of AI-based unmanned aerial vehicles (UAVs) because of the need for increased autonomy and reliability in unpredictable scenarios^1–3^. UAVs are finding applications in an ever-expanding range of fields. In addition to well-known uses such as video recording, surveillance, crop monitoring, security, and transportation, many new possibilities are emerging, including autonomous fieldwork (fertilization, mineral level measurement), package delivery, and mapping^4,5^.

Machine training has revolutionized various domains by enabling systems to adapt, optimize, and make intelligent decisions on the basis of data. From image recognition and natural language processing to autonomous driving and robotics, neural networks have demonstrated remarkable capabilities in solving complex problems. In control systems, machine training techniques have been applied to increase adaptability, improve fault tolerance, and optimize performance in dynamic environments^6^. Given these advancements, researchers have increasingly explored their integration into UAV control, leveraging neural networks to enhance stability, precision, and responsiveness^7,8^. Rather than directly replacing traditional control laws, some approaches use neural networks as supplementary components to refine and adapt control strategies. For example, in^9^, an RBF neural network functions as an online compensator for a proportional-integral-derivative (PID) controller, continuously training and adjusting to changing flight environment conditions. Similarly , an evolving type-2 quantum fuzzy neural network (eT2QFNN) has been employed alongside a PD (proportional-derivative) controller, forming a parallel control scheme that dynamically adjusts the control parameters. The proposition of combining model predictive control with a neural network in series to form a control system for trajectory tracking is presented in^10^. This solution, together with a disturbance observer, can adapt to and overcome any external disturbances that may affect the flight of the UAV. In another instance, the authors utilized the neural network as the observer for the single online approximator (SOLA)-based optimal control of a helicopter^11^. An interesting subsequent work^12^ presented a hierarchical structure of a neural network-based system aimed at planning the flight trajectory of a solar-powered UAV operating in near-space conditions to reduce energy demand and maximize solar energy gains. A complex system, where the preliminary control signal from the Lyapunov function is refined with a signal from a radial basis function neural network , resulting in a solution that can compensate for external disturbances, is presented in^13^. The authors of^14^ proposed a hybrid dual-scale neural network model, that integrates the generalized regression multimodel and cubature information filter (GRMM-CIF) framework, which has been shown to effectively track and predict the trajectories of complex maneuvering UAVs. The paper^15^ presented a novel control method that combines an artificial neural network (ANN) with a PD controller. This hybrid approach is designed to handle challenging scenarios involving fast flight, agile maneuvers, and motor failures, demonstrating its effectiveness in real-time applications. The authors of^16^ proposed a new vehicle dynamics model based on a time-delay feedback neural network. This model is designed to overcome the limitations of traditional vehicle dynamics models, which often rely on simplified physical principles that do not accurately reflect real-world conditions,

Another group of applications , in which neural networks are used in the context of flight control is multi-UAV cooperation. In the following work^17^, the authors consider trajectory and power optimization for cached UAV wireless networks, where deep neural networks are utilized for content recharging demand. The paper^18^ addresses the urgent problem of formation control for UAVs in unstable environments by proposing a novel approach using an adapted Kohonen neural network with various distance metrics. This method integrates intelligent geometric control principles to enable precise and flexible UAV movement along predefined routes, accounting for wind loads and potential vehicle collisions.

Finally, we consider applications, where the neural network is used as a direct source of the control signal for the UAV. The work^19^ presented an adaptive nonlinear controller for UAVs that addresses unknown time-varying disturbances and model parametric uncertainty. By using an adaptive neural network (NN ) to approximate a partially unknown system, the controller tracks a point along the UAV’s vertical body axis, avoiding the conventional two-subsystem control approach. The NN weights are updated online via a Lyapunov synthesis-based adaptive law, ensuring uniformly ultimately bounded tracking and adaptation errors. Reference^20^ proposes a two-layer structure for quadcopter UAVs, with leaders receiving tracking information and followers obtaining data via a graph theory-based network. A distributed formation-containment (FC ) control method is developed, which uses neural networks to handle uncertainties and the finite-time stability theorem to ensure effective tracking. Reference^21^ addresses transition control for a ducted fan VTOL ( vertical take-Off & landing) UAV and proposes a neural network-based controller to learn system dynamics and reduce tracking error. The authors derive a nonlinear system model and implement a novel control scheme featuring a projection operator, a state predictor, and adaptive input. The work in^22^ addresses standoff tracking, guiding a UAV to maintain a constant-height circular path over a moving target. The authors propose a Lyapunov guidance vector (LGV ) field for trajectory planning and a deep neural network (DNN) - based model predictive control (MPC ) scheme for tracking. The study presented in^23^ proposed a trajectory tracking control method for a quadcopter UAV via multidimensional taylor networks (MTNs ) and sliding mode robust control. It addresses realistic disturbances, including external interference and f-based parameter uncertainties. The approach decouples the system into position and attitude subsystems, utilizing MTNs for nonlinear compensation with reduced computational complexity compared with neural networks. A subsequent article^24^ proposed a novel nested control strategy using adaptive radial basis function neural network (RBFNN ) and supervised neural network (NN ) control with integrator backstepping (IBS ) for robust trajectory tracking of a quadcopter UAV. It effectively addresses modeling uncertainties, sensing noise, and external disturbances . Reference^25^ introduces a quadcopter UAV system employing neural-network enhanced dynamic inversion control with a sigma-pi neural network ( SPNN ) compensator. It addresses uncertainties in UAV dynamics, payload, and the environment, demonstrating improved performance in both simulation and experimental tests via high-precision optical motion capture. Compared to conventional proportional-integral-derivative control systems SPNN effectively reduces model and tracking errors , ensuring higher accuracy in attitude and trajectory control under varying flight conditions. Another interesting work is presented in^26^ - a novel trajectory tracking algorithm for quadrotor UAVs that combines the sigma-pi neural network with the backstepping control method. This integration aims to enhance the performance of quadrotor control systems, particularly in trajectory-tracking tasks. Reference^27^ proposed intelligent robust tracking control designs specifically for both uncertain holonomic and nonholonomic mechanical systems. This is a critical advancement as it addresses the complexities involved in controlling these types of systems under uncertainty.

The introduction of a neuro-fuzzy bee colony optimization method to optimize the membership function distribution in neuro-fuzzy controllers for quadcopter UAVs is presented in^28^. The study applies an adaptive neuro-fuzzy inference system (ANFIS) controller controlled by bee colony optimization (BCO) to track the quadrotor’s 2D trajectory effectively. Comparative results show that the proposed ANFIS-BCO method outperforms the ANFIS and PID controllers in trajectory tracking performance. Reference^29^ presented a fuzzy adaptive control law FACL ) for trajectory tracking of a low-scale UAV via a new fuzzy adaptive neural proportional-integral-derivative controller (FANPID ). The fuzzy adaptive control law (FACL) uses adaptivity from the FANPID-Lyapunov controller to estimate rotation angles on basis of reference trajectories. Reference^30^ employed fuzzy logic and neural network controllers for trajectory tracking of a UAV using the Parrot AR Drone 2.0 . A square-shaped reference trajectory is utilized in simulated and real-time experiments, with input signals derived from deviations and their derivatives in the *x* and *y* directions. Neural network parameters are updated online via variable structure systems theory, showing robust performance against disturbances. The trajectory regulating model reference adaptive controller (TRMRAC ), which uses a self-regulated intermediate reference model to enhance stability and robustness during adaptation, is presented in^31^. When implemented on a robotic arm with neural network-based dynamics approximation, TRMRAC demonstrates strong stability in the presence of unmodeled dynamics and input saturation. When tested on a quadcopter for quaternion attitude tracking, it outperforms traditional adaptive control methods. The authors in^32^ proposed a data-driven adaptive filtering algorithm that enhances tracking accuracy for highly maneuvering UAVs. This algorithm uses a recurrent neural network (RNN) - based motion model, which is trained on realistic simulated data from a medium fidelity Simulink model of a fixed-wing UAV. An active disturbance rejection control (ADRC) method is presented in^33^. By utilizing the tunicate swarm algorithm (TSA), the optimally parameterized ADRC significantly enhances the dynamic performance of the controlled tricopter. An interesting utilization of neural networks is when they are used as a system to navigate the UAV through visual input. As an example, we can distinguish the work presented in^34^, where the proposed system uses deep training techniques to enhance the visual navigation capabilities of UAVs. By employing a Gaussian smoothing function, the system preserves the main features of visual images, which is crucial for accurate navigation.

Despite the many advancements in combined neural network-classic control law systems, they still face significant limitations. Traditional control laws often rely on specific assumptions that can restrict the flexibility of neural networks. Moreover, these hybrid solutions introduce a more complex structure, making implementation on dedicated computing platforms more challenging. If not properly designed or configured, such systems may even underperform compared with traditional control methods. Additionally, the inherent complexity of hybrid systems can negatively impact their stability, leading to unpredictable behavior or unexpected performance issues. In contrast, relying solely on neural networks for control eliminates these challenges entirely.

This paper highlights the utilization of a neural network as a control algorithm to directly control the position-tracking of a holonomic UAV, such as a quadcopter. This is the main novelty of the research since there are no similar works about such usage of neural networks. Notably, at this moment neural networks overcome the advantages of traditional approaches to position tracking control, but they may turn out to be more elastic and cover a wider range of scenarios and applications depending on available data about control and measurement signals. Using neural network approaches to be treated as bioinspired and with the development of the technology first robots controlled by AI will be able to be seen. The presented approach involves implementing a position-tracking algorithm based on artificial potential fields across a series of simulations. These simulations encompass a wide range of trajectory shapes and speeds for the tracked point, aimed at generating extensive and relevant datasets. These datasets are crucial for effectively training and validating the neural network, ensuring robust performance in real-world applications of UAV trajectory control. The contributions of the presented research are as follows:

1. applying an artificial potential field as a reference method to generate a static (offline) training dataset for neural networks for different shapes of flight paths and speeds of the tracked point,
2. different well-known types of neural networks are trained and training results are evaluated with the use of obtained training data,
3. verify the position tracking effectiveness of different neural networks via flight simulations, for scenarios not included in the training data,
4. evaluation of the calculation cost for each neural network as a determinant of effectiveness in a real-world application, where real-time computing is crucial.

On the basis of the achieved research results, position tracking efficiency, and computing costs related to the network structure, it will be possible to identify neural networks, which will be used in further work to experimentally verify the research results with the usage of a Parrot drone and the same AR Drone toolbox, enabling control from MATLAB by Wi-Fi. Unitization of other models of UAV or mobile robot types requires repeated research because training data are dependent on specific features of the dynamics model of the drone.

## Model of a holonomic UAV

An important element of the simulation experiment is the proper model of the object considered. In this work , we consider the holonomic UAV in the form of the AR Drone 2.0 (quadcopter) , which can be expressed as a rigid and symmetric body with six degrees of freedom ( DoF). The holonomic nature of quadcopter UAVs means that the number of independent degrees of freedom equals the number of available control inputs. Thus, it can move in any direction without kinematic constraints. The equations utilized in the mathematical model of the quadcopter use two reference frames. The first is the body frame, denoted with the letter *B*, whereas the second is the global frame, marked with *G*.

To enable swift switching between the frames, the relationship between the two aforementioned frames can be established using the rotation matrix *R*, as shown in Eq. (1)^35,36^.

1 $$\begin{aligned} R= \begin{bmatrix} \text {sin}(\phi ) * \text {sin}(\psi ) - \text {sin}(\theta ) * \text {sin}(\phi ) * \text {sin}(\psi ) & - \text {sin}(\psi ) * \text {sin}(\phi ) - \text {sin}(\phi ) * \text {sin}(\theta ) * \text {sin}(\psi ) & \text {sin}(\theta ) * \text {sin}(\psi ) \\ \text {sin}(\theta ) * \text {sin}(\psi ) * \text {sin}(\phi ) + \text {sin}(\phi ) * \text {sin}(\psi ) & \text {sin}(\phi ) * \text {sin}(\theta ) * \text {sin}(\psi ) - \text {sin}(\phi ) * \text {sin}(\psi ) & - \text {sin}(\psi ) * \text {sin}(\theta ) \\ \text {sin}(\phi ) * \text {sin}(\theta ) & \text {sin}(\phi ) * \text {sin}(\theta ) & \text {sin}(\theta ) \end{bmatrix} \end{aligned}$$

where : $\phi$, $\theta$, and $\psi$ - are the roll, pitch, and yaw angles, respectively, which represent the angles between the axes of the body frame and the inertial frame.

The acceleration of the quadcopter is influenced by thrust, gravity, and linear friction, and depends on its mass. Combining these factors allows us to form an equation of the quadcopter’s linear motion defined in the inertial frame as in Eq. (2)^35,36^.

2 $$\begin{aligned} m \ddot{x}=\begin{bmatrix} 0 \\ 0 \\ -mg \end{bmatrix} -R \cdot T_{B}-F_{D} \end{aligned}$$

where : *m* - is the mass of the quadcopter ; *g* - is the gravitational acceleration ; $\ddot{x}$ - is the linear acceleration of the quadcopter ; *R* - is the rotation matrix ; $T_{B}$ and $F_{D}$ - are the thrust and drag forces in the body frame, respectively.

When calculating the thrust, we can derive the following formulas expressed in the body frame^35,36^:

3 $$\begin{aligned} T_{B}=\begin{bmatrix} 0 \\ 0 \\ F_{R}+F_{L}+F_{F}+F_{B} \end{bmatrix}=k \cdot \begin{bmatrix} 0 \\ 0 \\ \omega ^2_{R}+\omega ^2_{L}+\omega ^2_{F}+\omega ^2_{B} \end{bmatrix} \end{aligned}$$

where : $\omega _{i}$ - is the angular speed of the respective motors (subscripts: *F* -forward ; *B*-back, *R*-right, *L*-left) ; and *k* - are constants corresponding to the propulsion system (motor and propeller).

On the basis of fluid dynamics, we can derive the following equation describing the drag force on each axis of the inertial frame:

4 $$\begin{aligned} F_{d}= \frac{1}{2} \cdot \rho \cdot C_{D} \cdot A \cdot V^2 \end{aligned}$$

where : $\rho$ - is the density of the fluid (in this case, air) ; $C_{D}$ - is a dimensionless constant; *A* - is the propeller’s cross-section ; and *V* - is the linear speed.

To simplify the process of modeling the quadcopter, we can modify the Eq. (4). As a result, we obtain the following output^35,36^:

5 $$\begin{aligned} F_{D}=\begin{bmatrix} -k_d \cdot \dot{x}\\ -k_d \cdot \dot{y} \\ -k_d \cdot \dot{z} \end{bmatrix} \end{aligned}$$

where : $k_{d}$ - is the drag coefficient ; $\dot{x}$, $\dot{y}$, and $\dot{z}$ - are the linear speeds on each axis of the inertial frame.

The rotational equations of motion may be derived through Euler’s dependencies for rigid body dynamics^35,36^:

6 $$\begin{aligned} I \cdot \dot{\omega } + \omega \times \left( I \cdot \omega \right) = \tau _{B} \end{aligned}$$

where : *I* - is the inertia matrix ; $\omega$ - is the angular velocity vector ; and $\tau$ - is the vector of the external torques in the body frame.

Since the symmetrical rigid body of the quadcopter can be represented as two uniform rods intersecting at the origin of the body frame, with masses concentrated at each end (corresponding to the motors), the inertia matrix is expressed as follows^35,36^:

7 $$\begin{aligned} I=\begin{bmatrix} I_{xx} & 0 & 0\\ 0 & I_{yy} & 0 \\ 0 & 0 & I_{zz} \end{bmatrix} \end{aligned}$$

where : $I_{xx}$, $I_{yy}$, and $I_{zz}$ - represent the inertia on each axis.

The vector of external torques may be expressed as follows^35,36^:

8 $$\begin{aligned} \tau =\begin{bmatrix} \tau _{\phi }\\ \tau _{\theta } \\ \tau _{\psi } \end{bmatrix} = \begin{bmatrix} k \cdot \left( \omega ^{2}_{R} \cdot L - \omega ^{2}_{L} \cdot L \right) \\ k \cdot \left( \omega ^{2}_{F} \cdot L - \omega ^{2}_{B} \cdot L \right) \\ b \cdot \left( \omega ^{2}_{R} + \omega ^{2}_{L} - \omega ^{2}_{F} - \omega ^{2}_{B} \right) \end{bmatrix} \end{aligned}$$

where : *b* - represents a constant with suitable dimensions (related to drag-induced torque) ; and *k* - is a constant determined by the particular motor and propeller configuration.

Equation (6) can be reformulated as follows^35,36^:

9 $$\begin{aligned} \dot{\omega } = I^{-1} \cdot \left( \tau _{B} - \omega \times \left( \omega \cdot I \right) \right) \end{aligned}$$

By substituting the inertia matrix from Eq. (7) and the external torque vector from Eq. (8) into Eq. (9), we arrive at the final form of the rotational equation of motion^35,36^:

10 $$\begin{aligned} \dot{\omega }=\begin{bmatrix} \tau _{\phi } \cdot I^{-1}_{xx}\\ \tau _{\theta } \cdot I^{-1}_{yy}\\ \tau _{\psi } \cdot I^{-1}_{zz} \end{bmatrix} - \begin{bmatrix} \frac{I_{yy} - I{zz}}{I_{xx}} \cdot \omega _{y} \cdot \omega _{z} \\ \frac{I_{zz} - I{xx}}{I_{yy}} \cdot \omega _{x} \cdot \omega _{z} \\ \frac{I_{xx} - I{yy}}{I_{zz}} \cdot \omega _{x} \cdot \omega _{y} \end{bmatrix} \end{aligned}$$

Equations (2) and (10) form the dynamic model of the quadcopter, which can be utilized in numerical simulations. A complete model of the quadcopter in the state space form can be found in the following reference^37^. In our study, we employed a prebuilt model from the AR Drone 2.0 library available in MATLAB/SIMULINK R2024b .

## Artificial potential field method as a source of training data

The development of a control based on a neural network requires adequate data for the training process. Such data collection can be performed in several ways, starting from manual drone control and tracking predefined flight paths, and ending with the simulation of another control algorithm that is efficient in position-tracking applications. The algorithm used to produce the training dataset must use outputs typical for the control of the applied UAV and inputs supported by available hardware, i.e., onboard sensors and a tracked target localizing system. The research uses the quadcopter model defined for the Parrot drone in the AR Drone 2.0 toolbox for MATLAB. Because the Parrot drone is not an open UAV platform in which the onboard sensor system can be modified, it is assumed that the coordinates of the tracked target can be obtained from the local positioning system.

The Parrot drone control inputs are as follows: the desired roll and pitch angle, yaw rate, and vertical velocity. All inputs are limited to the range from $-1$ - 1. Available outputs to monitor the drone’s state or to close control loops are angles of roll, pitch, yaw, and linear speeds in three axes in the initial reference coordinate frame. The linear speeds can be used to compute the Parrot drone coordinates in the reference frame. Information about the actual roll and pitch angles does not matter in guiding the drone toward the tracked position; thus, only the yaw angle , which is the heading angle, is crucial. The set of required signals is finally defined , i.e. control inputs of roll, pitch, yaw rate, and vertical speed , followed by monitoring outputs, i.e. drone position as integrals of linear speeds, linear speeds in the x- and y- axes, yaw angle, and target position. It is intended to be obtained from the local positioning system or generated virtually.

One of the easiest methods that can be used in tracking applications with the use of the inputs and outputs listed above is the artificial potential field method. The method uses reversed gradients of artificial potential function to define the velocity vector field, which guides the robot toward the goal. It and the robot were objects with opposite magnetic potential field signs. It can also be used in position-tracking applications. Then, the point of a potential function minimum is located at the coordinates of a target point to be tracked by a quadcopter. Velocity vectors are given as reverse gradients of the artificial potential function pointing to the point of minimum ; thus, they can be used to guide a drone toward the target. The attraction potential field must be symmetrical to the minimum, and the most popular form is given by following the equation^38^:

11 $$\begin{aligned} U(x_R,y_R,z_R)=\left( \left( x_R -x_T \right) ^2+\left( y_R -y_T \right) ^2+\left( z_R -z_T \right) ^2 \right) \end{aligned}$$

where : $x_R$, $y_R$, $z_R$ - are the coordinates of the robot’s (quadcopter’s) position ; and $x_T$, $y_T$, $z_T$ - are the coordinates of a point to be tracked.

The gradients of the potential function 11 are defined as follows:

12 $$\begin{aligned} \nabla U(x_R,y_R,z_R)=\begin{bmatrix} 2\cdot \left( x_R-x_T \right) \\ 2\cdot \left( y_R-y_T \right) \\ 2\cdot \left( z_R-z_T \right) \end{bmatrix} \end{aligned}$$

A velocity vector field built with the gradients 11 also includes the saturation of the relative speed, i.e. the maximum length of the gradient (12). It cannot be greater than the saturation value of $V_{max}$. The velocity vector field is given by:

13 $$\begin{aligned} V(x_R,y_R,z_R)= \Biggl \{\begin{matrix} -\nabla U(x_R. y_R, z_R) \text { for} \\ \left| \nabla U(x_R. y_R, z_R) \right| \le V_{max}\\ -\frac{V_{max}}{\left| \nabla U(x_R. y_R, z_R) \right| }\cdot \nabla U(x_R. y_R, z_R) \text { for} \\ \left| \nabla U(x_R. y_R, z_R) \right| > V_{max} \end{matrix} \end{aligned}$$

where : $V_{max}$ - is the saturation value of the $\nabla U(x_R. y_R, z_R)$ gradient’s length ; and $\left| \nabla U(x_R. y_R, z_R) \right|$ - is the length of the gradient from 12.

**Fig. 1:**
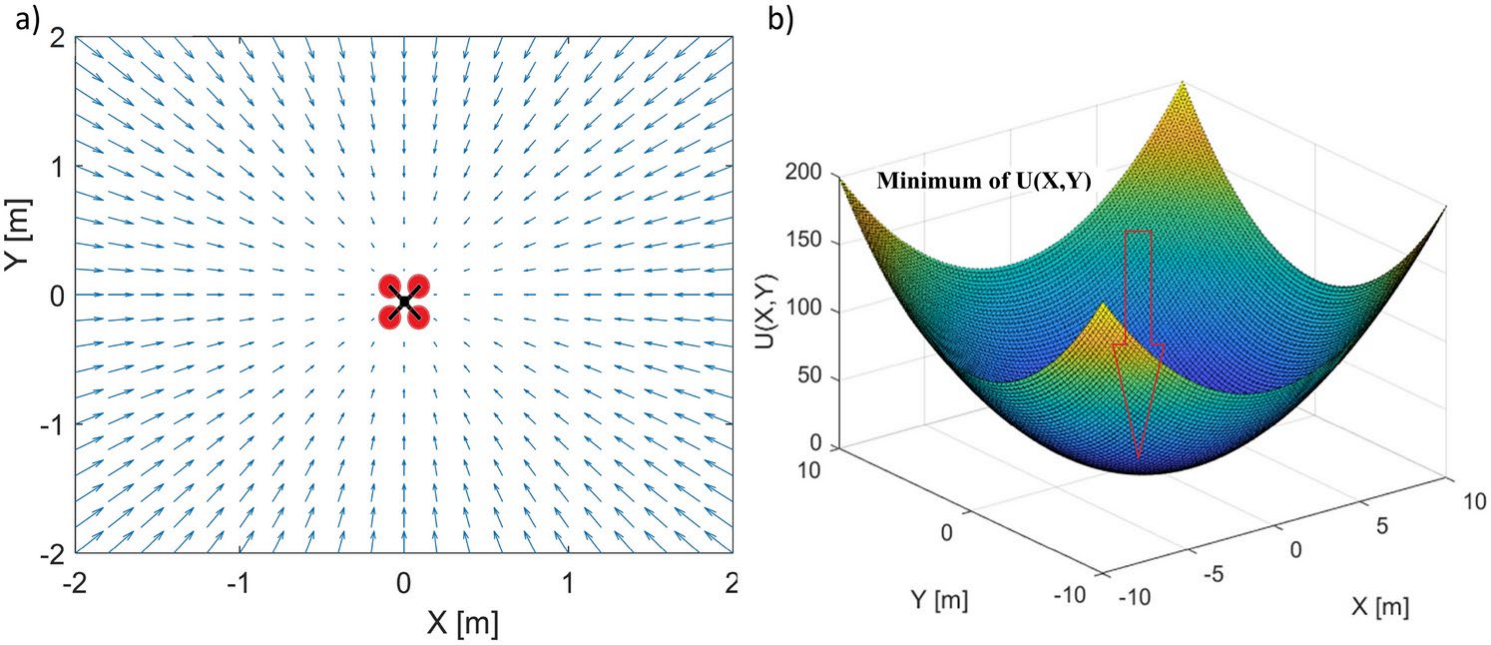
2D case of an artificial potential field (**a**) and the related field of velocity vectors (**b**).

A plot of the artificial potential function (11) is presented in Fig. 1 for a 2D case, along with the corresponding field of velocity vectors (13). A minimum point of the potential function is indicated by an arrow at the center of Fig. 1a. The field of velocity vectors is defined in a local coordinate frame fixed to the point of minimum. The pose and length of the velocity vectors result from the geometrical relationships between the minimum and its surroundings. Velocity vectors can be used to guide the quadcopter toward the point of minimum, which can be considered stationary in the local coordinate frame.

**Fig. 2:**
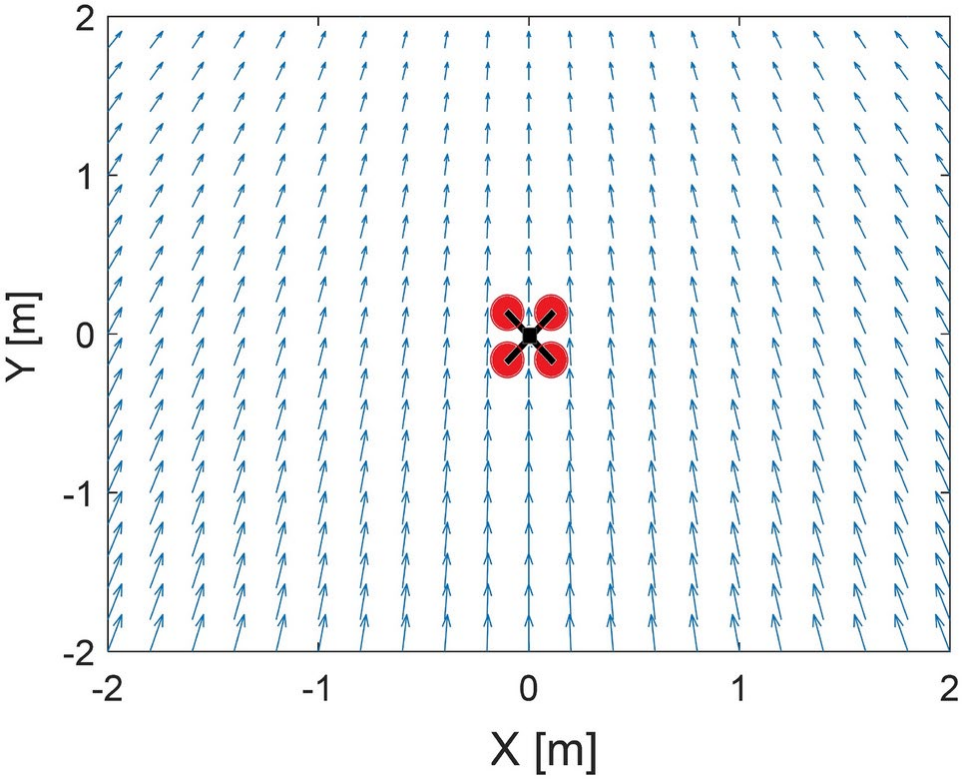
The superposition of the field of velocity vectors (13) and the vector $V_{TR}$ in the global coordinate frame.

Let us consider that the minimum point is moving with a velocity vector $V_{TR}$. Now, the field of velocity vectors that guide the quadcopter can be considered in the global coordinate frame as a superposition of the field of velocity vectors 13 and the vector $V_{TR}$. It will look like in Fig. 2.

**Fig. 3:**
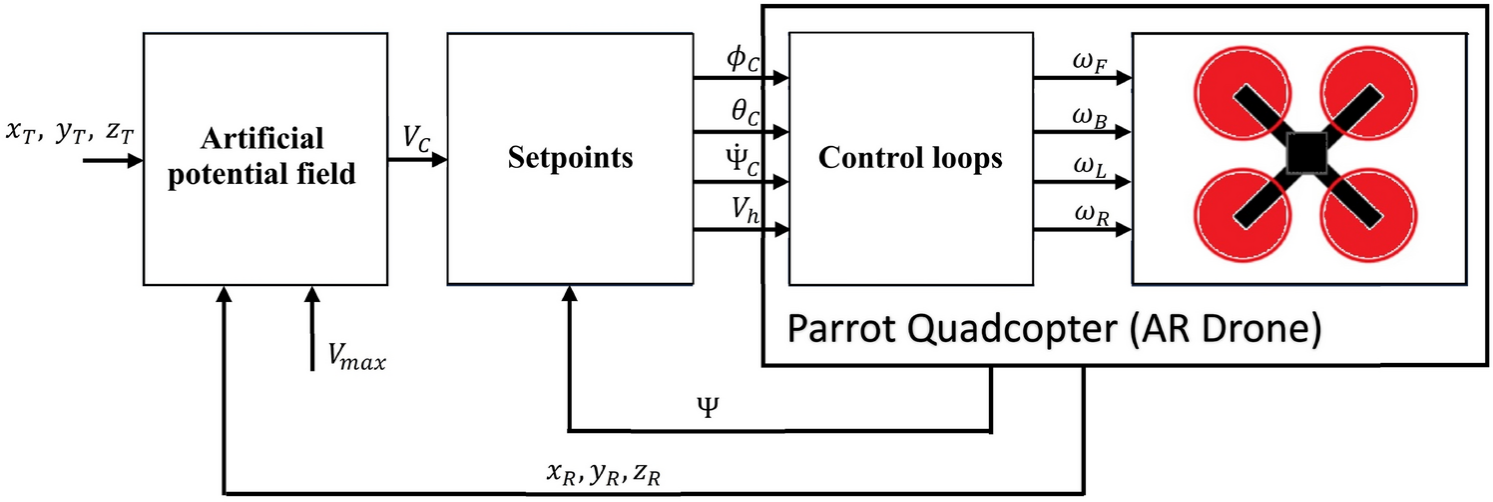
The plot of the superposition of the field of velocity vectors (13) and the vector $V_{TR}$.

The vector *V*, which is based on the gradient of the potential function, is responsible for minimizing the tracking error. Vector *V* can be used to obtain setpoint values for the quadcopter’s control inputs. Therefore, all necessary control laws must be designed to transform the control vector into quadcopter control inputs. A diagram of the control system and signal flow used is shown in Fig. 3.

The artificial potential field block inputs are the coordinates $x_T$, $y_T$, and $z_T$ of the point to be tracked, the actual coordinates of the quadcopter, i.e., $x_R$, $y_R$, and $z_R$ ; and $V_{max}$ - the maximum length of the $\nabla U(x_R, y_R, z_R)$ gradient as a coefficient. The setpoint block calculates control signals of roll $\Phi _C$, pitch $\theta _C$, rate of yaw $\Psi _C$, and vertical speed $V_h$ based on the velocity vector *V* and the value of actual yaw (heading) $\Psi$. The block of control loops controls flight with the use of the calculated setpoints. The equations implemented by this block are given below.

The Parrot drone’s linear speeds in the forward and longitudinal directions are controlled by the pitch and roll angles. Therefore, the desired roll and pitch angle values should be obtained from the vector *V*. First, it is necessary to determine the bearing to the tracked point given in the quadcopter’s body frame. The vector *V* must be rotated around the z-axis of the global coordinate frame by the current heading angle $\Psi$ of the quadcopter (Eq. 14). The x-axis and y-axis components of the vector $V_{Cr}$ can then be used to determine the required bearing (Eq. 15).

14 $$\begin{aligned} V_{Cr}= \begin{bmatrix} \text {sin}(\Psi ) & \text {sin}(\Psi ) & 0 \\ - \text {sin}(\Psi ) & \text {sin}(\Psi ) & 0 \\ 0 & 0 & 1 \end{bmatrix} \cdot V \end{aligned}$$

where : $\Psi$ - is the quadcopter’s current heading angle ; and *V* - is the velocity vector from Eq. (13).

15 $$\begin{aligned} \Psi _{B}=atan2\left( V_{Cr}(y), V_{Cr}(x) \right) \end{aligned}$$

where : $\Psi _B$ - is the bearing to the tracked point ; $V_{Cr}(x)$ and $V_{Cr}(y)$ - are the components of the vector $V_{Cr}$ (Eq. 13).

When both the vector $V_{Cr}$ and the bearing $\Psi _B$ are calculated, the required setpoints for the roll and pitch control loops can be designated. The AR Drone 2.0 model has defined ranges of desired roll and pitch angles from $-1$ - 1. These values correspond respectively to longitudinal linear speeds ranging from $-3.78$ to 3.78 m/s and transverse linear speeds ranging from $-2.88$ to 2.88 m/s . Therefore, the resultant maximum horizontal speed is approximately 4.75 m/s . In turn, the maximum vertical speed is approximately 0.88 m/s, which is related to the control signal $V_h=1$. These ranges of control inputs should be considered in calculations of the desired roll and pitch angles, which is why the equations defining the desired roll and pitch angles are as follows:

16 $$\begin{aligned} & \phi _C=\frac{\left| V_{Cr}(x,y) \right| }{2.88}\cdot \text {sin} (\Psi _B) \end{aligned}$$

17 $$\begin{aligned} & \theta _C=\frac{\left| V_{Cr}(x,y) \right| }{3.78}\cdot \text {cos} (\Psi _B) \end{aligned}$$

where : $\Psi _B$ -is the bearing to the tracked point ; $\left| V_{Cr}(x,y) \right|$ - is the length of the x-y plane projection of the vector $V_{Cr}$, which is limited to a range of $<0,2.88>$.

The ratios of $\left| V_{Cr}(x,y) \right|$ and 2.88 for $\phi _C$, and of $\left| V_{Cr}(x,y) \right|$ and 3.78 for $\theta _C$ represent the gain magnitudes independent of longitudinal and transverse speed control. In this research, it was assumed that the range of $\left| V_{Cr}(x,y) \right|$ is $<0, 2.88>$, thus the maximum horizontal longitudinal and transverse speeds become identical.

The vertical speed $V_h$ is simply the z-axis component of the vector *V*, and is limited to the range $<-1.0, 1.0>$.

18 $$\begin{aligned} V_h=\Biggl \{\begin{matrix} k_{V_h}\cdot V(z) \text { for } k_{V_h}\cdot V(z)\le 1 \cap k_{V_h}\cdot V(z)\ge -1)\\ 1 \text { for } k_{V_h} \cdot V(z)>1)\\ -1 \text { for } k_{V_h} \cdot V(z)<-1) \end{matrix} \end{aligned}$$

where : $k_{V_h}$ - is a gain coefficient that regulates the climbing rate.

The yaw rate, which regulates the rotation speed of the quadcopter around the *z*-axis of the global frame, is a function of the heading error, i.e., the difference between the actual heading of the quadcopter $\Psi$ and the heading defined by the vector *V*. The yaw rate signal equation is as follows:

19 $$\begin{aligned} \dot{\Psi }_C=k_{\dot{\Psi }}\cdot \frac{\left( atan2(V(y),V(x)) - \Psi \right) }{\pi } \end{aligned}$$

where : $k_{\dot{\Psi }}$ - is a gain coefficient ; $\Psi$ - is the actual heading of the quadcopter ; *V*(*y*), *V*(*x*) - represents the components of the vector *V* ; and $(\text {atan2}(V(y), V(x)) - \Psi )$ - heading error in the range $<-\pi , \pi>$.

All the required quadcopter control signals, which are designated from the velocity vector *V*, are given by Eqs. (14–19). These control signals are outputs of the neural network which implements the position-tracking control for the UAV. Let the outputs of the neural network be defined as a vector *Y* and the inputs as a vector *C*. Then both vectors are given by the equations below.

20 $$\begin{aligned} Y=\left[ \begin{matrix} \phi _C\\ \theta _C \\ \dot{\Psi }_C \\ V_h \end{matrix} \right] \end{aligned}$$

21 $$\begin{aligned} C=\left[ \begin{matrix} x_D\\ y_D\\ z_D\\ \Psi \\ \frac{\dot{x}_R}{3.78} \\ \frac{\dot{y}_R}{2.88} \\ D\\ \end{matrix} \right] \end{aligned}$$

where : $x_D$, $y_D$, and $z_D$ - are components of a unit vector originating at the UAV’s position and pointing to the target position ; *D* - is the normalized tracking error ; $dot{x}_R/3.78$ and $\dot{y}_R/2.88$ - are normalized longitudinal and traversal linear speeds of the UAV (ranges from -1.0 - 1.0) ; and $\Psi$ - is the actual heading of the UAV.

Let *D* be the distance between the UAV and the tracked point, which is given as follows:

22 $$\begin{aligned} D=\sqrt{(x_R-x_T)^2+(y_R-y_T)^2+(z_R-z_T)^2}, \end{aligned}$$

Then, the components of the unit vector from Eq. (21) are calculated via following:

23 $$\begin{aligned} \left[ \begin{array}{l} x_D\\ y_D\\ z_D \end{array} \right] =\left[ \begin{array}{l} \frac{x_T-x_R}{L} \\ \frac{y_T-y_R}{L}\\ \frac{z_T-z_R}{L} \end{array} \right] \end{aligned}$$

In turn, the normalized tracking error is given by:

24 $$\begin{aligned} E= \left\{ \begin{array}{l}\frac{D}{10}\text { for } D\le 10 \\ 1 \text { for } D>10 \end{array} \right. \end{aligned}$$

Equation (24) assumes that the maximum tracking error *E* observable for the neural network is 10 m , meaning that the control signals should reach their maximums above this value. The unit vector from Eq. (23) represents information about the relative position of the UAV and the tracked point, and $\Psi _C$, $\dot{x}_R/3.78$, and $\dot{y}_R/2.88$ define the current movement parameters of the quadcopter. Finally, *E* represents the tracking error in the $<0, 1.0>$ range. Therefore, the vector *C* from Eq. (21) collects all available information that can be used to produce training datasets for the neural network . It is simultaneously the input of the neural network.

## Neural network

For several years, artificial neural networks have been used in a wide range of applications. However, the term artificial neural networks includes several different structures, each of which has advantages as well as limitations. This article is limited to only feedforward networks, i.e. those in which the signal is passed from the neurons of the input layer through one or more hidden layers to the output layer. When designing a neural network, the number of neurons in the input and output layers is most often adjusted according to the dimension of the input and output space of the data held. Less often in the case of the input layer, selection or data extraction methods are used (e.g., principal component analysis)^39,40^. When individual inputs differ significantly in the range of values, preprocessing such as normalization or standardization is needed . The range of values of the input data is adjusted to the set of values of the activation function used in the output layer, or, as is often the case in a regression task, is left unchanged and the linear activation function is used.

The number of hidden layer neurons is still quite often selected by trial and error. Despite the limited efficiency and high time cost of such an approach, simplicity is favorable for this method. However, several methods exist for automatically selecting the number of neurons of the input layers, such as the activation function of neurons in the network or the training rate (in general, the hyperparameters of the neural network). Moreover, these methods are implemented in many popular software programs such as Statistica or MATLAB . One of the methods for optimizing neural network hyperparameters is the Bayesian optimization algorithm^41,42^. This algorithm is used wherever the objective function we want to optimize has a high computational cost, i.e. training the neural network. There are also several other more advanced algorithms such as reinforcement learning^43^, evolutionary algorithms^44,45^ or WASD Neuronet algorithms^46^.

An important problem during the training of a neural network is the phenomenon of overfitting, which is understood as the network’s overadaptation to the data from the training set while losing the ability to generalize the problem. The result of overfitting is the appearance of an error in the data from the testing set with a much larger value than was the case with the data from the training set. Consequently, many solutions are used to prevent overfitting of the neural network. The most commonly used solutions reduce the size of the neural network, which is understood as the number of modifiable parameters, and to use regularizers that prevent over-fitting to the data from the training set. Among these regularizers, methods such as L1, L2, early stopping, or dropout should be mentioned.

Notably, for neural networks with more hidden deep - layers artificial neural networks - an additional problem is the problem of vanishing gradients. This occurs when gradients decrease as they propagate backward through the individual layers of the neural network. This leads to slow convergence of the training process. The vanishing gradient problem can be solved by using rectified linear activation (ReLU ) and shortcut connections , among other methods. Skip connections are used in residual networks ( ResNets ), in which the signal is given to the next hidden layer and this layer is omitted .

## Results

Different shapes and sizes of trajectories to be tracked are used to train and validate designed structures of neural networks , such as regression multilayer perceptron network (MLP) with a single hidden layer having N neurons ($MLP\_1\_N$ ), a regression multilayer perceptron network with two hidden layers having M and N in each layer ($MLP\_2\_M\_N$), a deep neural network ( DNN ), and residual neural network ( ResNet). The selected shapes of the flight paths contain typical segments of the UAVs’ path, i.e. flying along a straight line, sharply turning approximately 90 degrees or more, and making circles around the target point. Thus, using rectangles, triangles, circles, and 8-shaped paths in the training dataset should provide enough information to ensure that the neural network can control UAVs flying through more complex flight paths. Dynamic environmental changes, such as wind disturbances were not considered in this research. Future work will involve extending the simulation model with noises to feedback signals, i.e. position and speed, with the use of wind gust models.

**Fig. 4:**
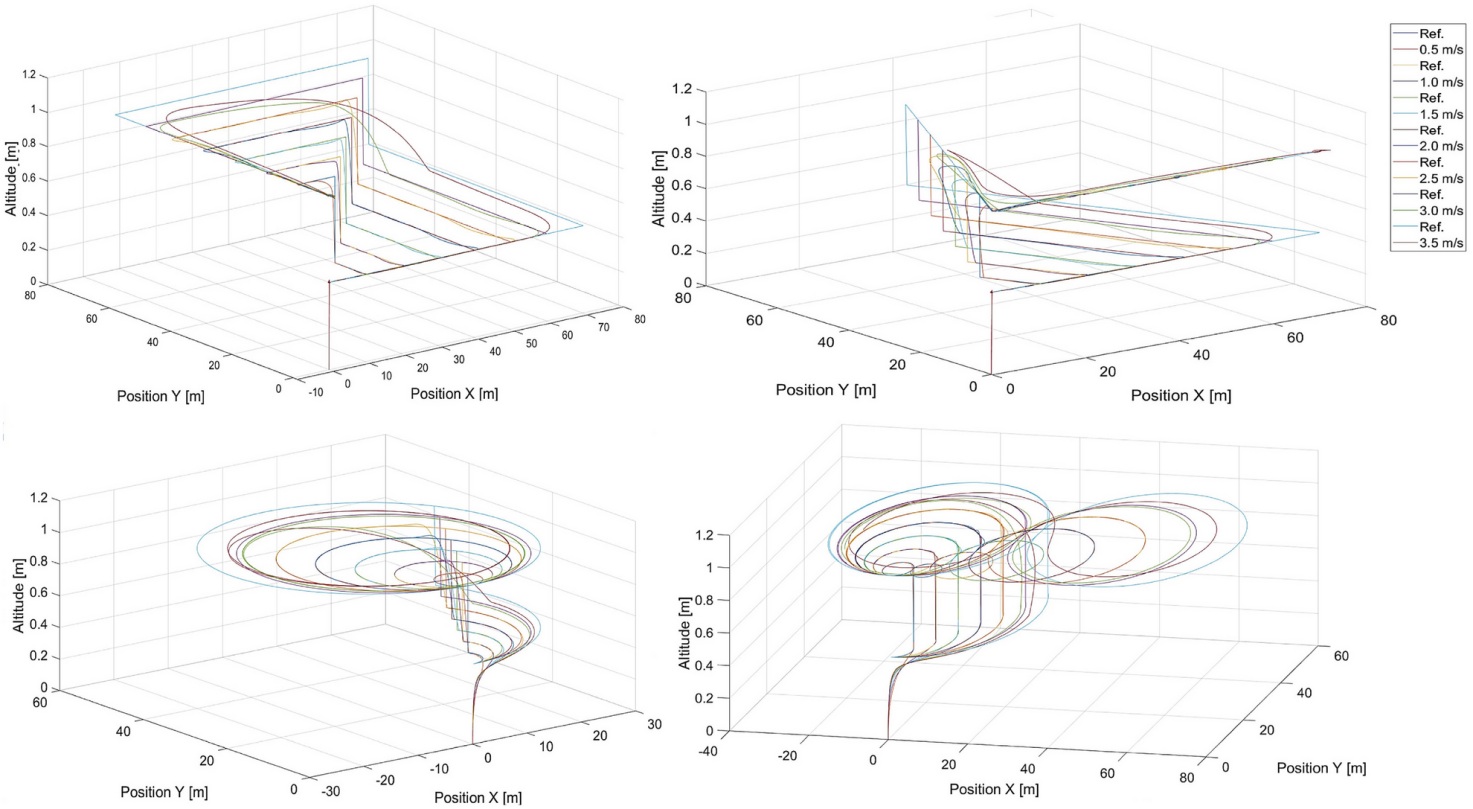
Training datasets 1-4 - rectangular, triangular, circular, and ’8’- shaped trajectories for different speeds of the tracked point, i.e. 0.5, 1.0, 1.5, 2.0, 2.5, 3.0, and 3.5 m/s.

The training trajectories are presented in Fig. 4 for different speeds , which are selected to evenly represent the range of speeds achievable by the UAV. The trajectories of the moving point to be tracked are labeled by ’Ref.’ in the legends. In these figures, there are also tracking flight paths of the quadcopter recorded while it is under the control of the artificial potential field control approach from “Neural network” section for different speeds of the tracked point, i.e. 0.5, 1.0, 1.5, 2.0, 2.5, 3.0 and 3.5 m/s . In the training process, different sizes of these trajectories are also applied, i.e. scaled by *x*0.5 and *x*2, but only for 0.5, 1.5, and 2.5 m/s , for all other cases, the scale is *x*1. Each case includes a step change in altitude. Data recorded during simulations of these trajectories are used as neural network inputs and outputs in the training process. The simulations are performed in MATLAB/Simulink software with the use of the AR Drone 2.0 toolbox for the Parrot quadcopter. The fundamental sample time is set to 0.065 s. The simulation stop time is at the 130th second (scale x1 - 28 flight paths), at the 65th second (scale x0.5 - 12 flight paths), and at the 260th second (scale x2 - 12 flight paths), which results in 28x2, 000, 12x1, 000, and 12x4, 000 samples of input vector *C* (21) and output vector *Y* (20) respectively . Finally, we obtain a total of 116, 000 samples in the dataset used in the training process. The dataset is split randomly into a training set ($85{\%}$ of the samples), and a validation set ($15{\%}$ of the samples). The same training dataset was used for all the neural network structures and no data augmentation techniques were applied.

Regression multilayer perceptron networks with a single-layer MLP ( $MLP\_1\_N$ ) were designed and trained in Statistica and with a double-layer MLP ($MLP\_2\_M\_N$) in MATLAB software. Other networks, such as deep neural networks ( DNNs ) and residual neural networks ( ResNets ), were developed and trained directly in MATLAB .

### Regression multilayer perceptron networks

#### Single-layer MLP

To find the best fit for the MLP neural network, an automatic search mode of the Statistica Data Mining module was applied. Only a range of the number of neurons in the hidden layer and types of activation functions for the hidden and output layers are defined. On the basis of preliminary neural network training iterations, the *logistic* activation function for both the hidden and output layers yielded the best results. Therefore, further research focused on finding the optimal number of neurons N in the hidden layer, with the range limited to between 100 and 300 neurons for the $MLP\_1\_N$ networks. Numerical flight simulations were performed to validate how the obtained neural networks work in position tracking control. The maximum achievable speed of the quadcopter in the simulations was $20{\%}$ greater than the speed of the tracked point (range of 0.5- 3.5 m/s). Thus, it was lower than the maximum speed performance of the Parrot AR Drone 2.0, i.e., approximately 4.75 m/s .

**Fig. 5:**
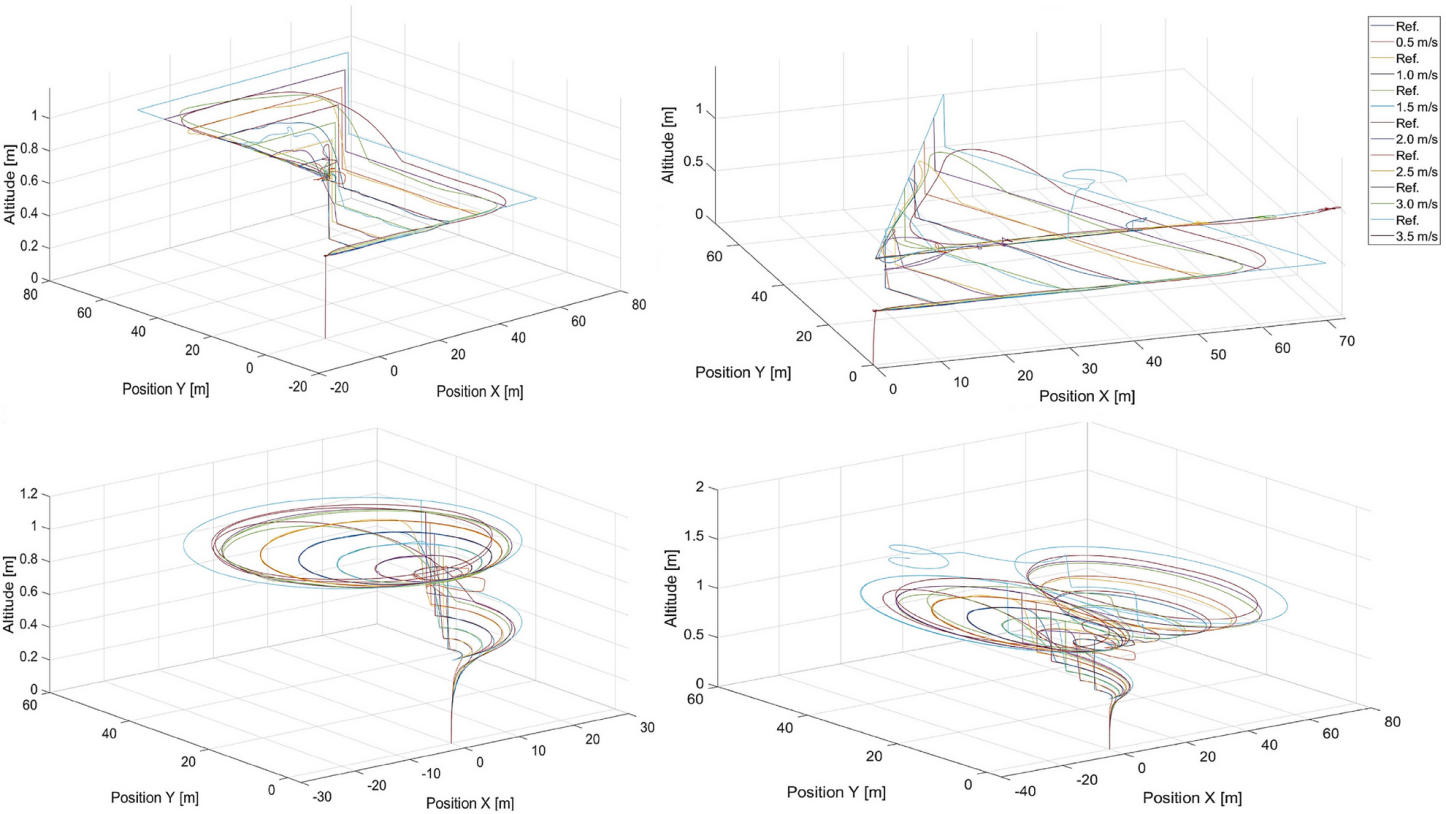
Simulated flight paths in rectangular, triangular, circular, and ’8’ - shaped trajectory tracking scenarios using control based on the $MLP\_1\_N$ neural network with 131 neurons for different speeds of the tracked point, i.e. 0.5, 1.0, 1.5, 2.0, 2.5, 3.0 and 3.5 m/s, where “Ref.” denotes reference trajectories for the given speeds.

**Fig. 6:**
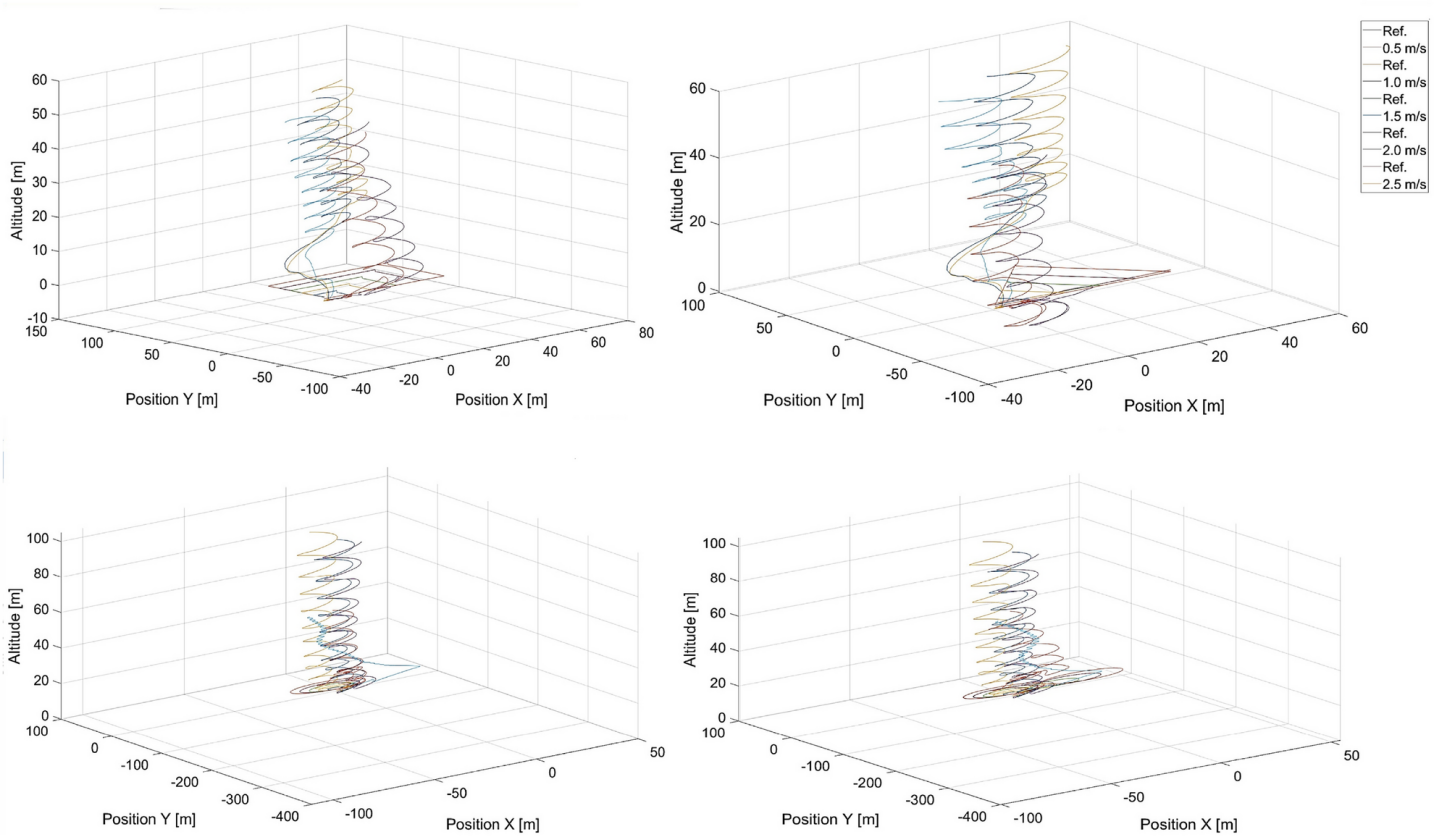
Simulated flight paths in rectangular, triangular, circular, and ’8’-shaped trajectory tracking scenarios using control based on the $MLP\_1\_N$ neural network with 277 neurons for different speeds of the tracked point, i.e. 0.5, 1.0, 1.5, 2.0, and 2.5 m/s, where “Ref.” denotes reference trajectories for given speeds.

Figures 5 and 6 present the response of the quadcopter position tracking control based on the $MLP\_1\_N$ neural networks with 131 and 277 neurons in the hidden layer, respectively.

Figure 5, shows that, generally, the quadcopter under the control of the $MLP\_1\_N$ neural network with 131 neurons can track the desired trajectory effectively. However, it fails in specific cases, such as the triangular trajectory at a speed of 2.0 m/s , the circular trajectory at a speed of 0.5 m/s , and the 8-shaped trajectory at speeds of 1.5 m/s and 2.0 m/s . Among the presented results, no consistent pattern or rule can be identified.

In turn, the results for the $MLP\_1\_N$ neural network with 277 neurons in the hidden layer are worse, as it fails in every trajectory shape and speed case. Therefore, there is no sense in making plots for all investigated speeds which could make the figure unclear. The results for $MLP\_1\_277$ are interesting because a neural network with a greater number of neurons in the hidden layer should theoretically yield better results, as shown in Table 1. The $MLP\_1\_131$ neural network also performs well for almost all cases, but a problem occurs when the tracked point stops . It is visible in a rectangular shape. For circular trajectory shapes, the radius of the turns is slightly smaller than that for the tracked point . This indicates that the UAV is unable to reach the speed of the tracked point.

**Table 1:** Results of training for MLP_1_N neural networks with different numbers of neurons in the hidden layer from Statistica. No—number of neurons, Trn. qty—training quality index (correlation), Vld. qty—validating quality index (correlation), Trn. err.—training error (from Statistica), Vld. err.—validating error (from Statistica).

No	Trn. qty	Vld. qty	Trn. err.	Vld. err.
MLP_1_109	0.993962	0.992964	0.000754	0.000762
MLP_1_126	0.993587	0.992957	0.000580	0.000587
MLP_1_131	0.995414	0.994340	0.000494	0.000561
MLP_1_135	0.994405	0.993427	0.000590	0.000611
MLP_1_176	0.993070	0.992337	0.000634	0.000648
MLP_1_213	0.996372	0.995315	0.000348	0.000395
MLP_1_235	0.994989	0.993870	0.000432	0.000467
MLP_1_277	0.997765	0.996589	0.000252	0.000321

All the training and validation quality indices are expected to increase with the number of neurons in the hidden layer, and the corresponding errors should decrease . Unfortunately, these changes are not monotonic. It appears that there are local minima that make it difficult to find a global minimum. The training algorithm is based on finding a minimum of SSE via the BFGS method (Broyden-Fletcher-Goldfarb-Shanno algorithm). In Statistica, the correlation between the target value and the predicted value is used as a quality qualifier for training and validation. The results from Table 1 contrast with those in Fig. 6. This discrepancy may result from a too-small training dataset while considering the unlimited number of variants of input values such as the relative position, orientation, path shape, and speed between the quadcopter and the tracked point. Indeed, the dataset used only 52 combinations of sequences of samples, i.e. 12x1, 000, 28x2000, and 12x4000 . The total number of 116, 000 samples in the dataset is not small, but it still refers to those 52 combinations. Thus, any disruption or error in numerical processing can cause the neural network to fail to find correct output values, similar to the effect of overfitting. Neural networks with higher indices of training and validation quality more precisely adapt to these specific 52 combinations, making them less robust to errors . They can be caused by numerical processing or other sources of disturbances that can occur even in simulations with the same parameters. Conversely, applying a greater number of flight scenarios in the dataset can result in an extremely long training process .

#### Double-layer MLP

For an MLP network with two hidden layers $MLP\_2\_M\_N$ , the number of neurons in the input and output layers was identical to the approach used for an $MLP\_1\_N$ network with a single hidden layer. The selection of hyperparameter values was performed via a Bayesian optimization algorithm. The algorithm indicated that the input data should be standardized, the activation functions of all the network layers should be hyperbolic tangents , and the numbers of hidden layer neurons should be 105 and 54 . On the basis of these results, a series of neural networks were trained with the above parameters and slightly modified parameters. training was carried out via the Broydon - Fletcher - Goldfarb - Shanno (BFGS) quasi-Newton method, and the number of training cycles was set at 5, 000 iterations. The results of a few selected examples of network training are shown in Table 2.

**Fig. 7:**
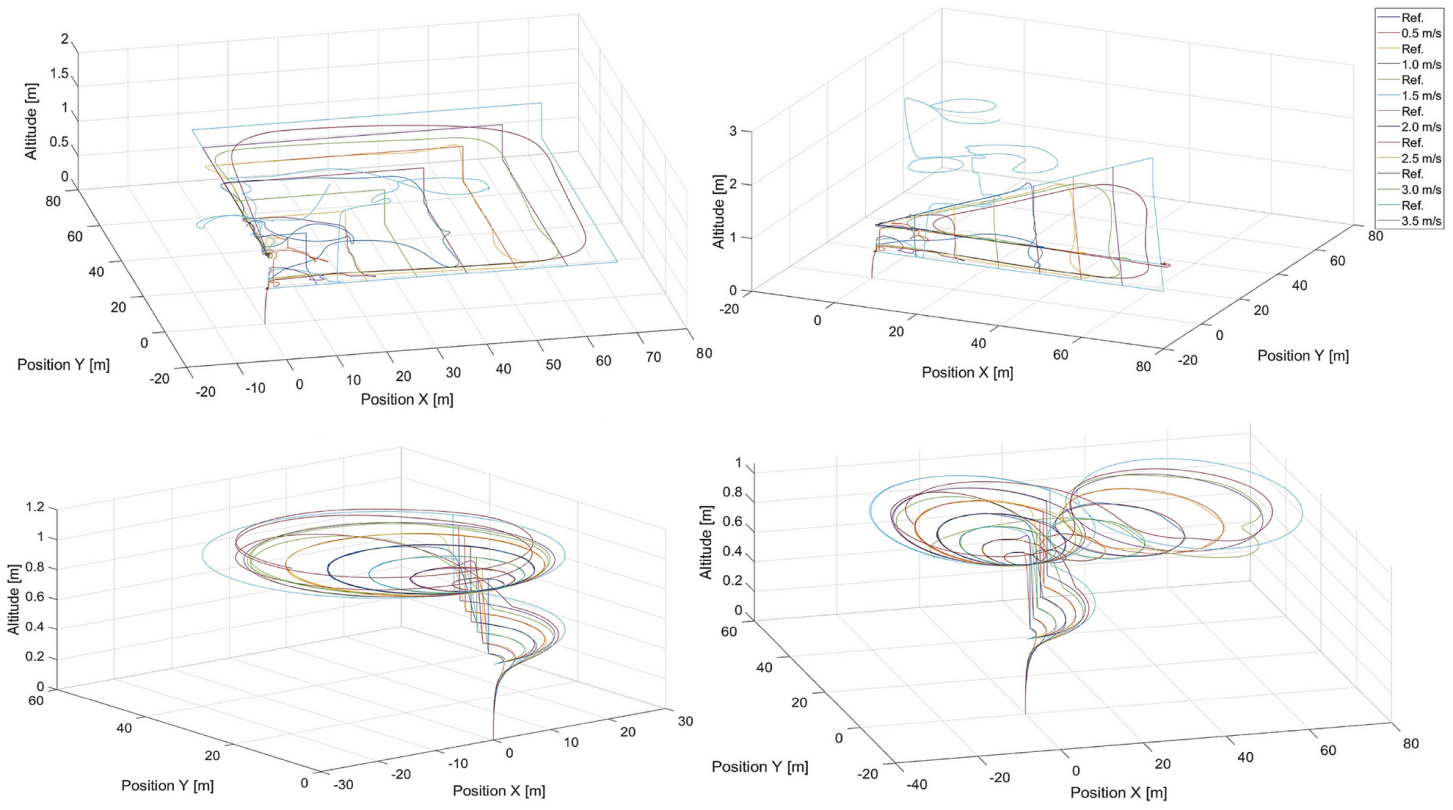
Simulated flight paths for all shapes of trajectories used in tracking scenarios applying control based on the $MLP\_2\_107\_54$ multilayer perceptron network with two hidden layers for speeds of the tracked point, i.e.: 0.5 m/s, 1.0 m/s, 1.5 m/s, 2.0 m/s, 2.5 m/s, 3.0 m/s and 3.5 m/s, where “Ref.” denotes reference trajectories for given speeds and shapes..

An analysis of the results shows that the network with fewer neurons in the hidden layers ( $MLP\_2\_280\_40$ ) obtained significantly poorer results than the other networks did . On the other hand, networks with several hidden neurons close to that the optimization algorithm considers to be the best have very similar quality. The specific number of neurons is less important here than the initial weights of the network, which were drawn independently from the network just before the start of training . The largest network tested ( $MLP\_2\_120\_60$ ) gave the best results on the training set and was only slightly weaker than the best results on the validation set. Since the generalization ability and neural network running time are the most important factors, this network was rejected. The $MLP\_2\_107\_54$ network, for which the lowest RMSE error on the validation set and the second-best correlation coefficient results were obtained, was selected as the best feedforward network with two hidden layers. Notably, the training time for all networks of this type was very long, and finding the best network architecture was cumbersome. The trajectories from the flight simulations for the $MLP\_2\_107\_54$ network are shown in Fig. 7.

**Table 2:** Results of training for $MLP\_2\_M\_N$ neural networks with different numbers of neurons in the hidden layers. No—the number of neurons in each layer, Trn. qty—quality index (correlation) for the training set, Vld. qty—validating quality index (correlation), Trn. RMSE—training error, Vld. RMSE—validating error.

No	Trn. qty	Vld. qty	Trn. err.	Vld. err.
MLP_2_80_40	0.9952	0.9956	0.03190	0.03207
MLP_2_105_54	0.9968	0.9975	0.02050	0.02283
MLP_2_106_55	0.9977	0.9971	0.01993	0.02228
MLP_2_107_54	0.9980	0.9972	0.01928	0.02091
MLP_2_120_60	0.9982	0.9971	0.01720	0.02109

### Deep neural networks

Neural networks with more than 2 hidden layers were used for deep training (DNN ). In each case, the input layer was a featureInputLayer with seven neurons, and then dense layers with a ReLU activation function with a variable number of neurons were used. In the last two dense layers, the sigmoid activation function was always adopted. After each dense layer, batch normalization was applied. The last layer - the output layer - consists of 4 neurons. In addition, to reduce the possibility of overfitting the network, dropout was applied to all the hidden layers. In this work, all DNNs used only dense layers (without using convolutional layers). Table 3 shows the results of training selected DNN models. The indications used *HID*1*x*.....*xHIDL*(*Nr*), *BVal* denote a neural network that has HID1 neurons in the first hidden layer and HIDL neurons in the last hidden layer. The number of neurons in the hidden layers occurring between HID1 and HIDL is twice less than in the previous layer. The value of Nr in parentheses indicates the probability of an element being zeroed during training (dropout). BVal, on the other hand, stands for batch size. For example, a 1600*x*..*x*100(0.1), *B*64 network describes a 7*x*1600*x*800*x*400*x*200*x*100*x*4(0.1) network which has the following architecture:
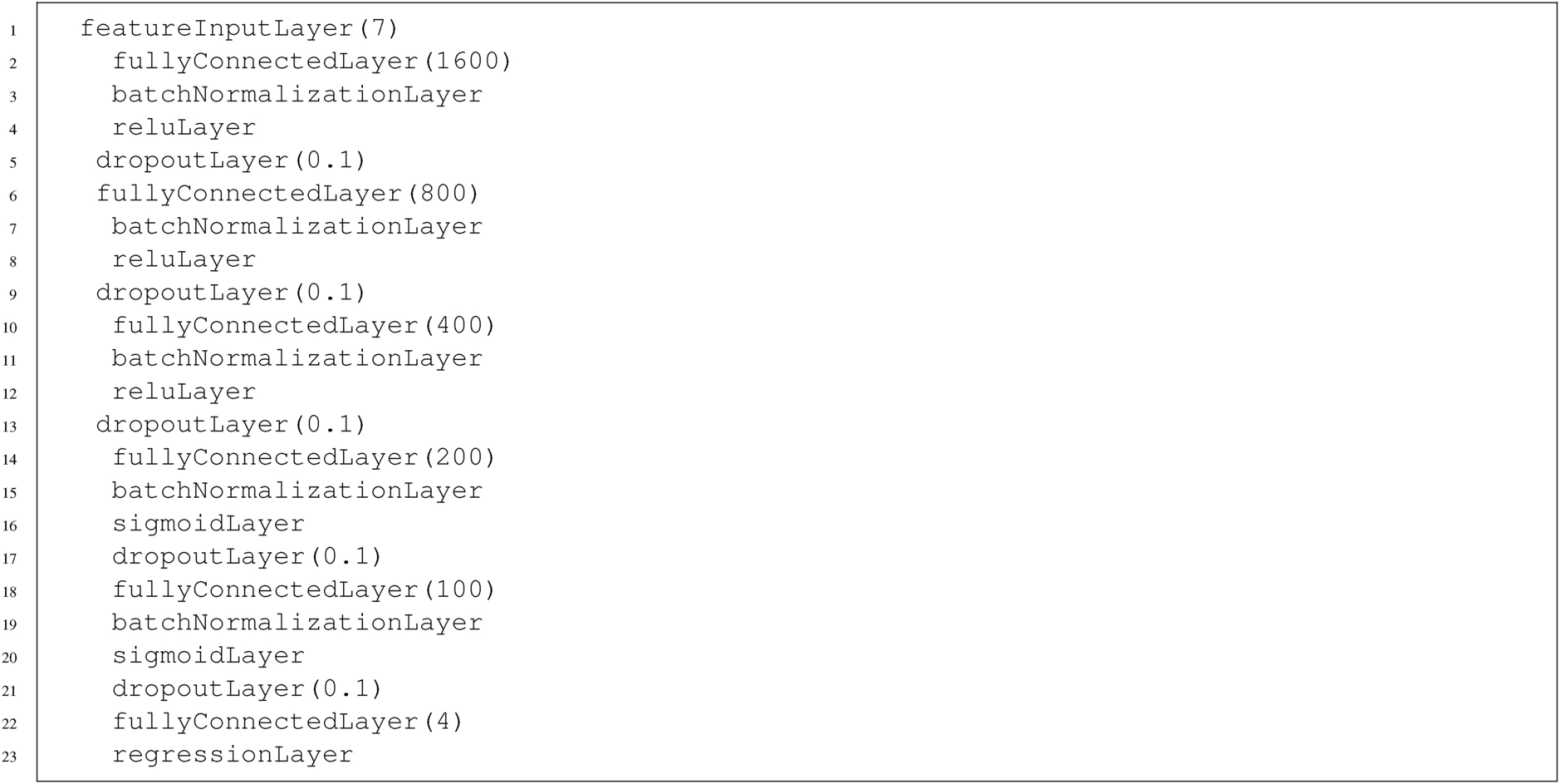


The well-known Adam algorithm was used to learn DNNs^47^. training was mostly done with parameters with standard values, with the number of epochs set to 30 and the batch size set to 64, 128, or 256. The results shown in the table3 indicate that the network learns best if the dropout is 0.1. In the case of batch size, it is noted that for networks with more neurons, better results are achieved for larger values of batch size. Two of the networks shown in the table achieve good training results. These are 1600x..x100(0.1), B64 and 3200x. . . x100(0.1),B256, and the differences between their results are insignificant. Therefore, the first network was chosen as the better one, as it has a smaller number of neurons. This network will be called *DNN*1 in the paper.

**Table 3:** Results of training for DNNs. No—the symbol of the deep neural network, Trn. qty—quality index (correlation) for the training set, Vld. qty—validating quality index (correlation), Trn. RMSE—training error, Vld. RMSE—validating error.

No	Trn. qty	Vld. qty	Trn. err.	Vld. err.
800x400x200x100(0.1), B64	0.9915	0.9922	0.03249	0.03271
1600x..x100(0.1), B64	0.9940	0.9941	0.02367	0.02421
3200x ...x100(0.1),B64	0.9926	0.9935	0.03090	0.03104
3200x ...x100(0.1),B128	0.9938	0.9940	0.02386	0.02479
3200x ...x100(0.1),B256	0.9939	0.9942	0.02341	0.02494
6400x ...x100(0.1),B128	0.9923	0.9928	0.03275	0.03309
2048x ...x16(0.1), B128	0.9936	0.9938	0.02488	0.02661

**Fig. 8:**
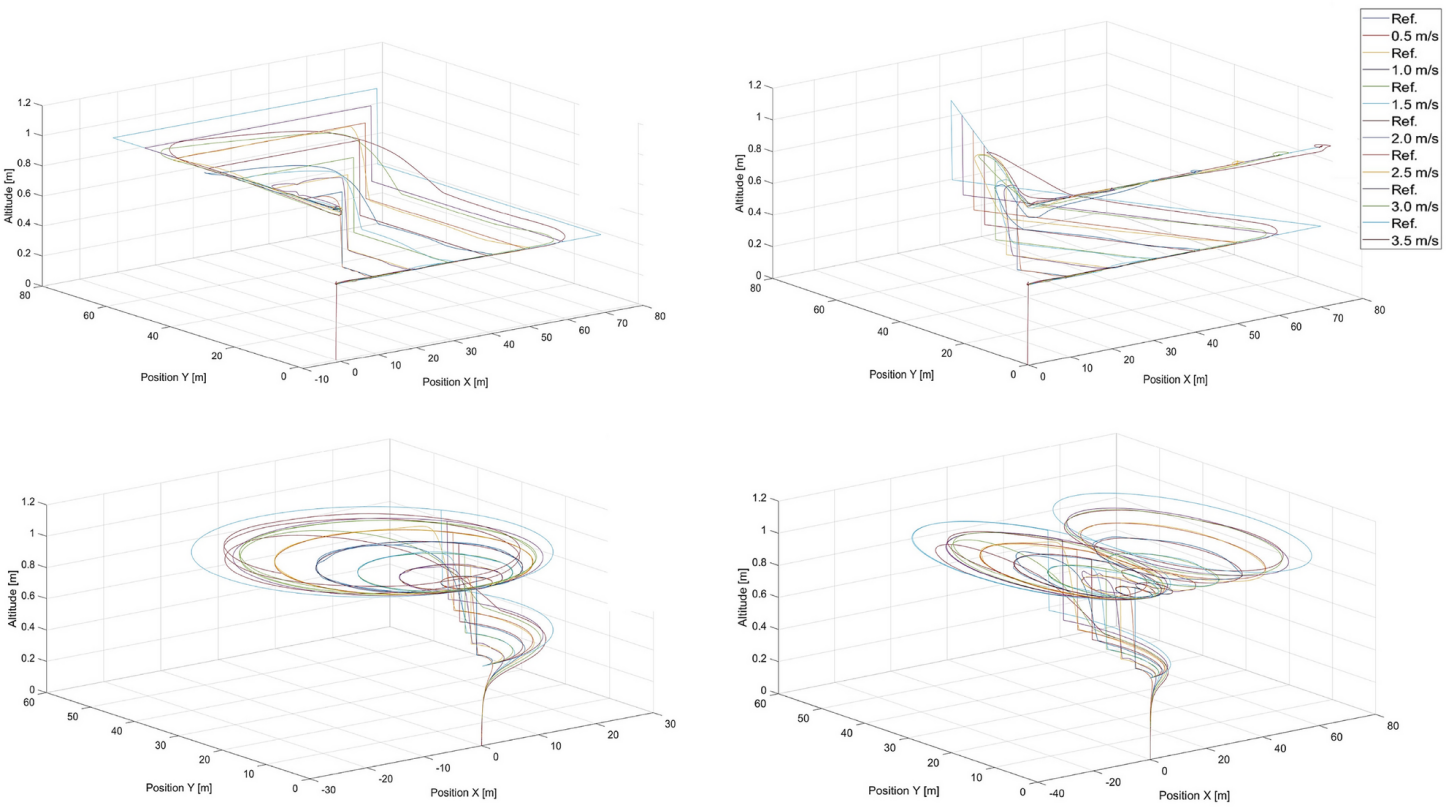
Simulated flight paths for all shapes of trajectories used in tracking scenarios applying control based on the *DNN*1 neural network for speeds of the tracked point, i.e.: 0.5 m/s, 1.0 m/s, 1.5 m/s, 2.0 m/s, 2.5 m/s, 3.0 m/s and 3.5 m/s, where “Ref.” denotes reference trajectories for given speeds and shapes.

The simulation results for flights with the applied DNN1 neural network are presented in Fig. 8. According to the trajectory curves of the quadcopter shown there, in general, the quadcopter under the control of the *DNN*1 neural network can follow the desired trajectory as accurately as possible. Inaccuracies in the mapping are apparent, particularly where the desired trajectory forms a right angle. This is, of course, impossible for the real system to reproduce, and it is good that this is mapped into the training results of the deep network. The problem with mapping these turns increases as the speed increases, and it is most visible at a speed of 3.5 m/s.

### Residual networks

**Fig. 9:**
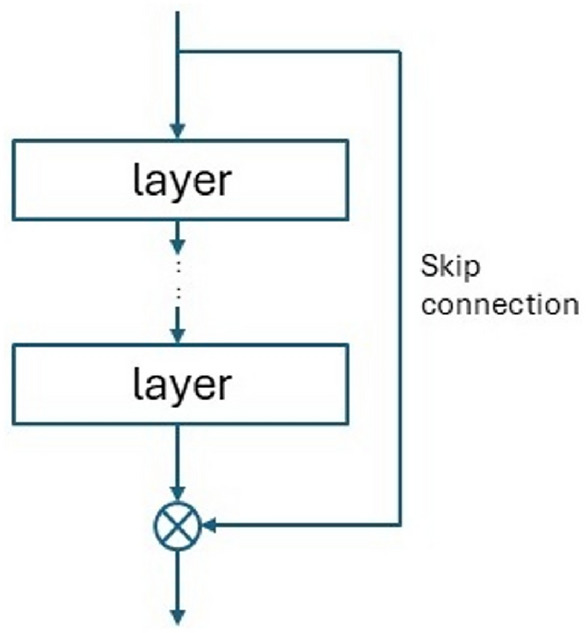
Residual unit.

A distinctive feature of residual networks (ResNets ) is the presence of residual units, in which the signal passes both through the neural network layer or layers (Fig. 9), and is transferred unchanged to its output (shortcut connection), where it is summed. ResNet can be considered as a stack of residual units. Although ResNet was originally applied to image processing and classification^48^, the idea can also be used in a regression task using only dense layers in the residual units. For example, the paper^49^ presents a ResNet model aimed at nonlinear regression. The authors show that the use of a deep residual network significantly improves the ability to model nonlinear regression compared to traditional approaches. Replacing convolutional layers with fully connected layers in residual blocks enables the model to better capture complex patterns in the data. The work of^50^ provides evidence that the ResNet architecture, with its proper training, is capable of improving accuracy in performing regressions on complex data sets. ResNet, thanks to the concept of residual learning, makes it possible to build deeper neural networks, which in the case of data with high variance and complexity is crucial for better results.

In the research covered in this paper, 3 to 8 residual units with a variable number of neurons were used. Each residual unit contained a dense layer with batch normalization and ReLU. In addition, to reduce the possibility of overfitting the network, a dropout equal to 0.1 was applied to all the hidden layers. The most interesting results are presented in Table 4. In this table, *NrR* stands for the residual block, which has one dense layer with *Nr* neurons. If more than one dense layer is used in a residual block, its number is given after *R*. The Adam algorithm with parameters such as those of DNNs is used to train ResNet .

**Table 4:** Results of training ResNet. *No*—the symbol of the deep neural network, Trn. qty—quality index (correlation) for the training set, Vld. qty—validating quality index (correlation), Trn. RMSE—training error, Vld. RMSE—validating error.

No	Trn. qty	Vld. qty	Trn. err.	Vld. err.
7x4096Rx ...x32Rx4	0.9806	0.9823	0.06349	0.06438
7x2048Rx ...x32Rx4	0.9797	0.9808	0.06044	0.06057
7x1024Rx ...x32Rx4	0.9818	0.9832	0.05286	0.05337
7x512Rx ...x32Rx4	0.9713	0.9763	0.05865	0.05901
7x1024R2x ...x32R2x16x4	0.9470	0.9528	0.07361	0.07481
7x512Rx512Rx512Rx512Rx4	0.9828	0.9831	0.05432	0.05499
7x800Rx800Rx800Rx800Rx4	0.9820	0.9829	0.05473	0.05671
7x400Rx400Rx400Rx400Rx4	0.9738	0.9763	0.07077	0.07191

The best results were achieved for the 7x1024Rx...x32Rx4 networks. Adding another residual unit with 2048 neurons, as well as removing the most numerous residual units worsened the results. Notably, adding more dense layers in residual units (7x1024R2x...x32R2x16x4 network) negatively affect the quality of the network. Interestingly, the highest correlation value for the training set was achieved for a network that had 4 residual units of 512 neurons in each dense layer. However, the quality of the validation set of this network is noticeably lower. Nevertheless, one could consider this network a fairly good alternative, offering a compromise between complexity and quality, of the best ResNet obtained.

**Fig. 10:**
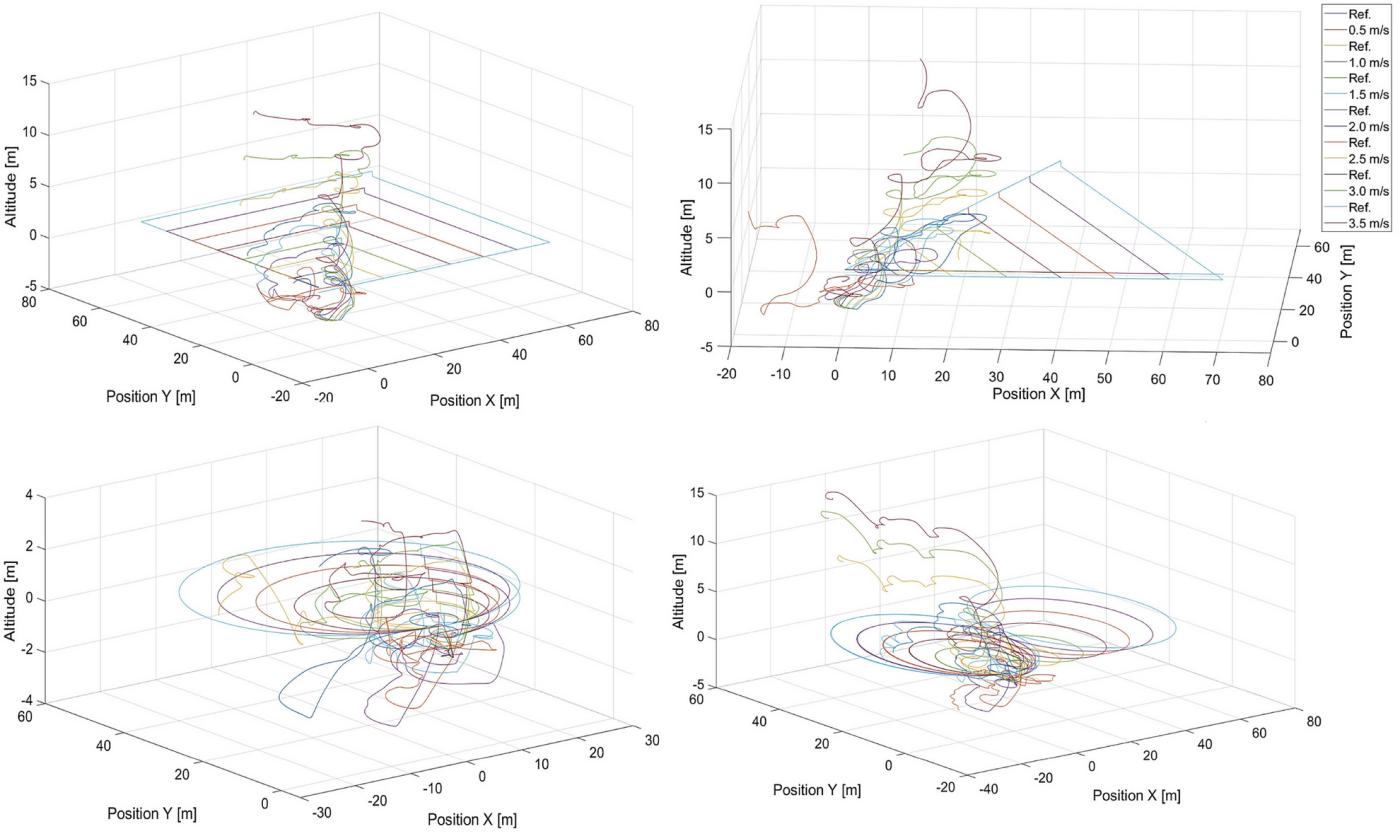
Simulated flight paths for all shapes of trajectories used in tracking scenarios applying control based on the ResNet residual neural network for speeds of the tracked point, i.e. 0.5 m/s, 1.0 m/s, 1.5 m/s, 2.0 m/s, 2.5 m/s, 3.0 m/s and 3.5 m/s, where “Ref.” denotes reference trajectories for given speeds and shapes.

Figure 10 shows the quadcopter’s flight paths when residual network ResNet was applied. The best-selected ResNet does not cope with trajectory reproduction, regardless of speed or shape. The problem stems from the fact that already at the beginning the value of the control given by the network varies so much that it gets lost and is later unable to return to the correct trajectory. Therefore, it was concluded that ResNet in this particular task does not achieve the correct level of approximation of the mapping of the phenomenon.

### Summary of training neural networks

To compare the training quality of different neural network architectures, the error generated by Statistica was converted to RMSE values . The best networks of each type were tabulated (Table 5). The results clearly show that the best network is the MLP with two hidden layers, whereas the worst is ResNet. The latter case, it is noteworthy for the very low value of the lowest validating correlation, which is less than 0.95 and significantly lower than the second-worst value of just under 0.98. It is worth noting, however, that the RMSE for ResNet is admittedly 2.5 times larger than that for MLP networks with two hidden layers (0.0534 vs. 0.0209), but overall the error is not large.

**Table 5:** Results of training for different types of neural networks i.e. MLP_1_131, MLP_2_107_54, *DNN*1 and ResNet neural networks from MATLAB. *Type*—type of neural network, the lowest training correlation—the lowest correlation coefficient among all four outputs, the lowest validating correlation—the lowest correlation coefficient among all four outputs, Trn. err.—training error (RMSE), Vld. err.—validating error (RMSE).

Type	The lowest training correlation	The lowest validating correlation	Trn. err.	Vld. err.
MLP_1_131	0.9878	0.9878	0.0319	0.0321
MLP_2_107_54	0.9941	0.9911	0.0192	0.0209
DNN1	0.9799	0.9805	0.0235	0.0243
ResNet	0.9444	0.9487	0.0529	0.0534

### Qualitative comparison of neural network on the basis of flight simulations

As indicated, the results from neural network training trials are not coherent with the flight control quality and effectiveness . This was proven in the case of $MLP\_1\_N$ with a single hidden layer or shown by differences between the results for $MLP\_2\_107\_54$ and *DNN*1 . Even if a regression multilayer perceptron network with a greater number of neurons in the hidden layer achieves better results i.e. smaller errors and higher correlation coefficients (given as Pearson’s correlation) , it becomes worse in flight simulations than it is in training with the use of the same training data. Therefore, two criteria were defined as follows to compare the results of the flight simulations. The first is the root mean squared error of position tracking errors D ($RMSE_D$), which assesses control effectiveness in reference trajectory tracking:

25 $$\begin{aligned} RMSE_D=\sqrt{\frac{1}{N}\cdot \sum _{n=0}^{N}D^{2}_{n}} \end{aligned}$$

where : *D* - is the distance between the UAV and the tracked reference point at the $n^{th}$ moment from Eq. (22) and *N* - the number of samples from the simulation (simulation duration of 130 s . divided by a sampling time of 0.065 s results in 2000 samples). The second criterion is the mean correlation between the coordinates of the UAV and the tracked point. It is given by the equation below:

26 $$\begin{aligned} r_{m}=\frac{1}{3}\cdot (r(x_R,x_T)+r(y_R,y_T)+r(z_R,z_T)) \end{aligned}$$

where : r- is Pearson’s linear correlation between random variables.

Tables 6 and 7 respectively contain the average values of the root mean squared errors ($RMSE_D$—Eq. 25) and the average correlation $r_{m}$ for all the shapes of the tracked trajectory used for all the investigated neural network configurations .

**Table 6:** Averaged values of $RMSE_D$ (Eq. 25) over speeds $V=0.5, 1.0, 1.5, 2.0, 2.5, 3.0$ and 3.5 m/s for different neural networks and shapes of the tracked trajectory shapes obtained from flight simulations, $MLP\_1\_N$—multilayer perceptron network with single hidden layers, $MLP\_2\_107\_54$—multilayer perceptron network with two hidden layers with a structure of 7x107x54x4, *DNN*1 and *DNN*2 - deep neural networks. *Avg*.—averaged $RMSE_D$ value over speeds and trajectory shapes.

NN	Square	Triangle	Circle	8-shaped	Avg.
MLP_1_119	10.9581	10.0989	0.8914	3.8455	6.4485
MLP_1_131	1.1408	1.3771	0.8928	1.5270	1.2344
MLP_1_135	2.9932	3.8404	2.3663	4.3839	2.3663
MLP_1_176	1.1148	1.2575	0.9011	2.2541	1.3819
MLP_1_293	1.1694	1.2410	3.6600	4.5778	2.6621
MLP_2_107_54	1.4091	2.1553	0.9082	1.3432	1.4539
DNN1	1.1189	1.2507	0.9424	1.0722	1.0960
DNN2	1.1247	1.2156	0.9270	1.0648	1.0830
ResNet	7.0918	6.7221	5.1454	6.0285	6.2470

**Table 7:** Averaged correlations $r_{m}$ (Eq. 26) over speeds V=0.5, 1.0, 1.5, 2.0, 2.5, 3.0 and 3.5 m/s for different neural networks and shapes of the tracked trajectory obtained from flight simulations, $MLP\_1\_N$—multilayer perceptron network with single hidden layers, $MLP\_2\_107\_54$—multilayer perceptron network with two hidden layers, *DNN*1 and *DNN*2 - deep neural networks. Avg.—averaged correlation value over speeds and trajectory shapes.

NN	Square	Triangle	Circle	8-shaped	Avg.
MLP_1_119	0.2756	0.3835	0.9555	0.7555	0.5925
MLP_1_131	0.9692	0.9516	0.9527	0.9222	0.9489
MLP_1_135	0.8755	0.7846	0.8763	0.6884	0.8062
MLP_1_176	0.9712	0.9600	0.9554	0.8545	0.9353
MLP_1_293	0.9519	0.9593	0.8377	0.6485	0.8494
MLP_2_107_54	0.9107	0.8573	0.9479	0.9566	0.9181
DNN1	0.9734	0.9610	0.9558	0.9594	0.9624
DNN2	0.9697	0.9639	0.9560	0.9579	0.9619
ResNet	0.3080	0.2195	-0.1664	0.2308	0.1480

On the basis of Tables 6 and 7, the lowest average ($RMSE_D$ - Avg.) and the highest average correlation coefficient ($r_{m}$ - Avg.) are achieved by the deep neural networks *DNN*1 (with a neural structure of 7x1600x800x400x200x100x4) and *DNN*2 (with a neural structure of 7x1600x800x400x200x100x4 ). The $MLP\_1\_131$ neural network ranks is not much worse than the methods others do , despite its simpler architecture with only one hidden layer and greater computational efficiency.

Interestingly, for specific trajectory shapes such as squares, triangles , and ’8’, the $MLP\_1\_176$ network yields higher correlation values than does $MLP\_1\_131$, which are close to those of *DNN*1 . This indicates that the UAV’s trajectory more closely resembles the reference trajectory. Nevertheless, $MLP\_1\_176$ remains an inferior solution compared with $MLP\_1\_131$ and the deep networks *DNN*1 and *DNN*2 .

A detailed analysis of the simulation results presented in both tables does not unequivocally identify a single network that consistently outperforms the others across all the scenarios . It can be assumed that any deep network will retain its superiority with more training data. The worst results are achieved by ResNet and $MLP\_1\_119$.

**Fig. 11:**
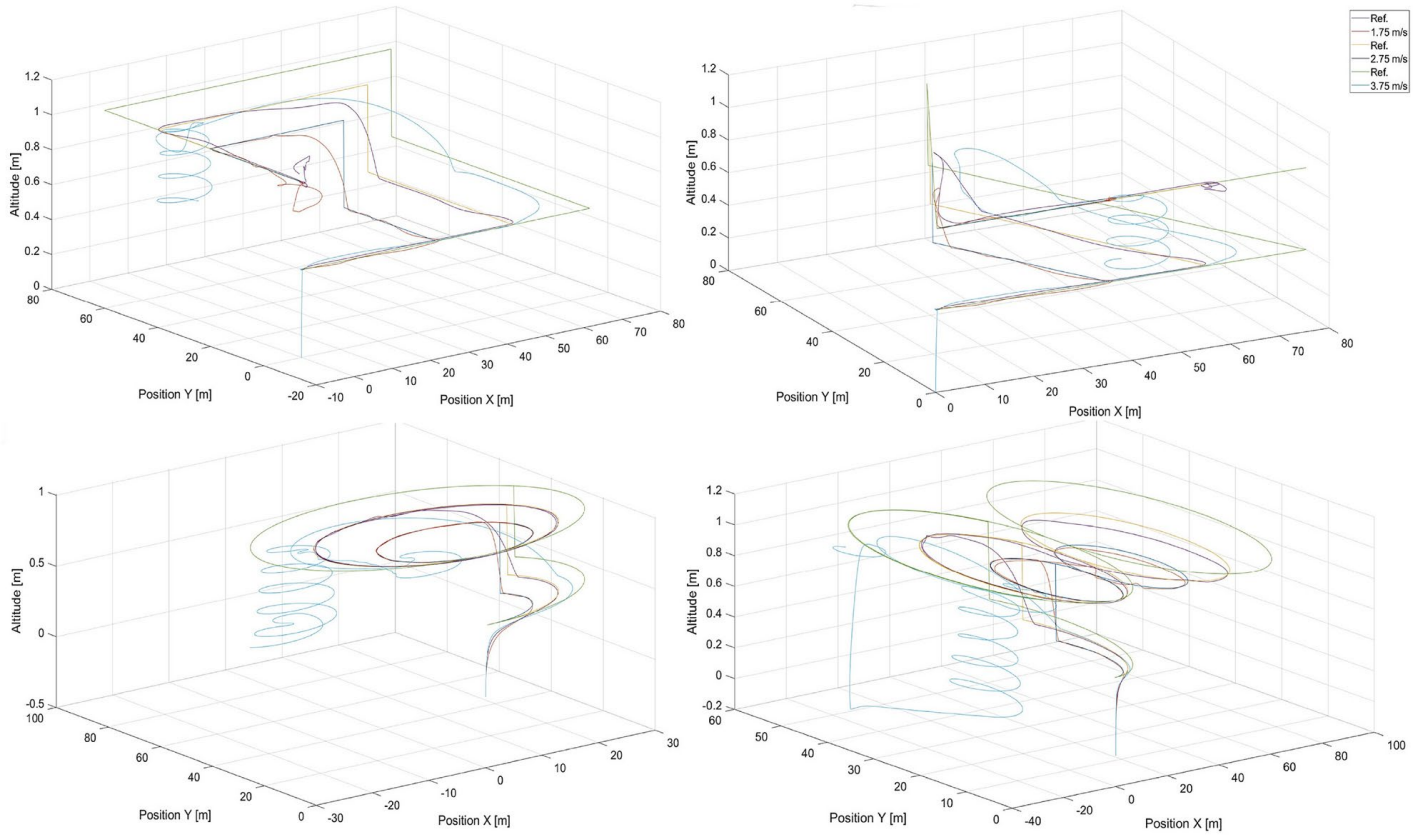
Simulated flight paths in the rectangular, triangular, circular, and the ’8’ - shaped trajectory tracking scenarios using control based on the $MLP\_1\_N$ neural network with 131 neurons for speeds were not used in the training dataset, i.e. 1.5, 1.75, 2.75, and 3.75 m/s, respectively. “Ref.” denotes reference trajectories for the given speeds.

**Fig. 12:**
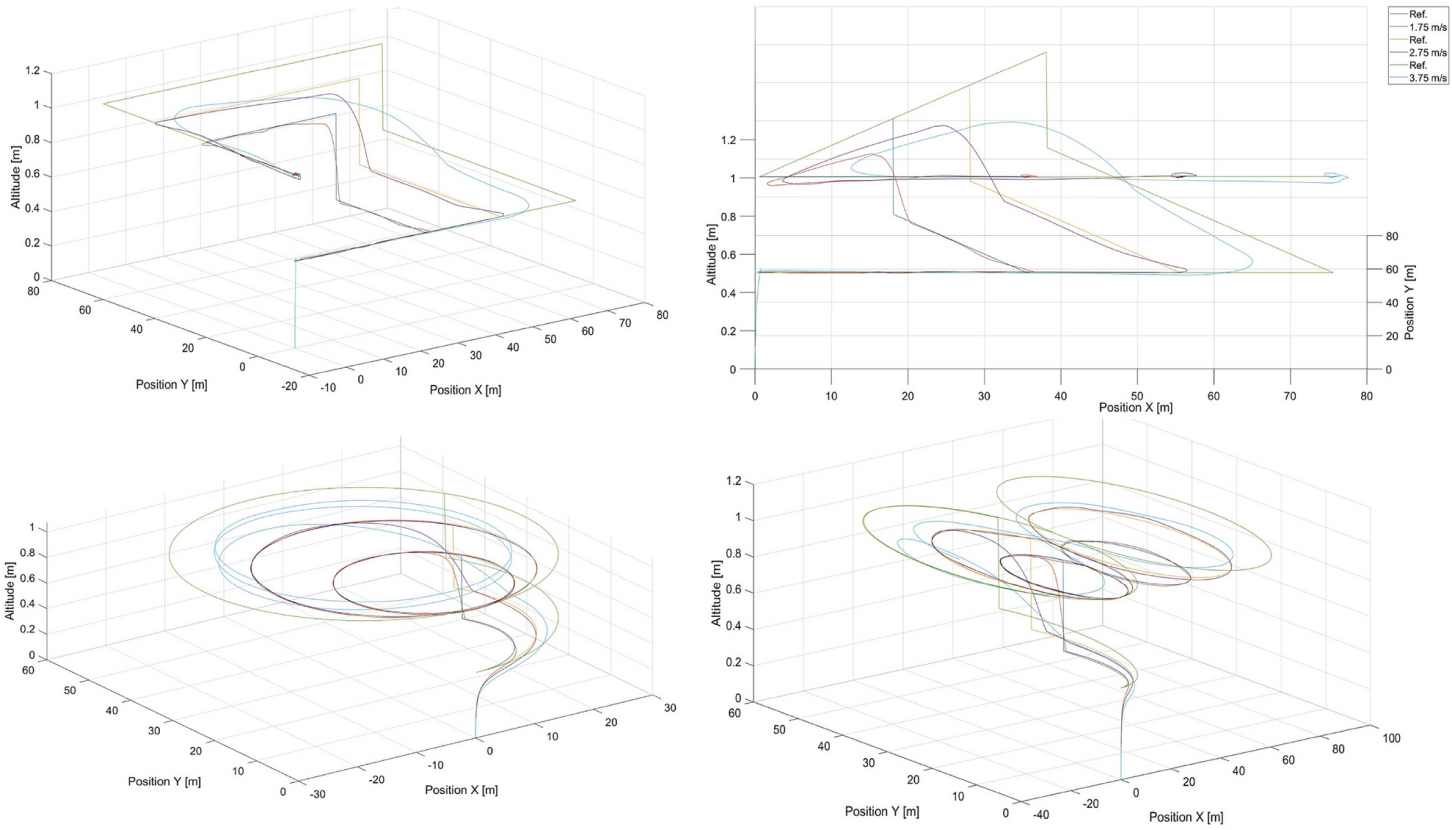
Simulated flight paths for all shapes of trajectories used in tracking scenarios applying control based on the *DNN*1 neural network for speeds were not used in the training dataset, i.e. 1.5, 1.75, 2.75, and 3.75 m/s, where “Ref.” denotes reference trajectories for the given speeds.

To validate the extrapolation capabilities of the neural networks in their considered usage in position tracking applications, simulations were performed for *DNN*1 and $MLP\_1\_131$, for speeds that were not used to compose the training dataset, i.e. 1.75, 2.75, and 3.75 m/s . Flight paths from these simulations are presented in Figs. 11 and 12 for $MLP\_1\_131$ and *DNN*1 respectively .

A comparison of both Figs. 11 and 12 reveals that the deep neural network offers a better capability for extrapolation than does the single-layer $MLP\_1\_N$ network. This is essential in our case , where the training dataset cannot cover the entire space of possible combinations of flight parameters or would have to be extremely large.

Interestingly, $MLP\_1\_131$ starts to fail after a specific amount of time and only for a speed of 3.75 m/s , which is outside of the speed range of 0.5 - 3.5 m/s. Most likely , during flight, the UAV is unable to reduce tracking error effectively by increasing its speed over the speed of the tracked reference point. The speed difference is too high, and the UAV begins making circles when the tracked point reaches its final position and stops. This is confirmed by the shortening of sharp turns by the UAV in the case of square and triangle paths. However, this effect is not present in the case of the *DNN*1 network and proves its effectiveness.

Notably, the neural network itself is a kind of black box, most often considered through the prism of the quality of the representation of the desired output signal. The structure of the network therefore makes it difficult to analyse exactly why one network is better than another. In this case, we are dealing with two networks that are similar in structure. Both are built exclusively of dense layers, with the main difference being the number of layers. The $MLP\_1\_N$ network used a logistic activation function, while the DNN1 network used a ReLU function. The computational complexity associated with the $MLP\_1\_N$ activation function is quite a bit higher, but this is offset by the much lower number of weight connections and neurons. The larger number of weights of the DNN1 network is an advantage, however, as it is thus able to learn a more complex mapping than $MLP\_1\_N$. The analysis of the results in Table 5 related to the RSME error obtained for the learning and validation sets indicates that the error of the $MLP\_1\_131$ network is quite higher than that of the DNN1 network. This suggests that the DNN1 network will obtain better simulation results than $MLP\_1\_131$. Nevertheless, this error is low enough that the $MLP\_1\_131$ network performs very decently in mapping trajectories whose velocities are within the range of velocities found in the learning sequence. It is also important to note that the DNN1 network is not overly deep and uses regularisation techniques that increase the generalisability and thus positively affect the extrapolability.

Finally, Table 5 presents correlation values, and the RMSE is based only on outputs of control signals in the training datasets and outputs generated by neural networks. Therefore, the values of the RMSE are lower than those in the Table 6. In the case of Table 6, the error $RMSE_D$ is defined as the distance between the reference point and the UAV. Therefore, it allows the assessment of trajectory tracking efficiency while Table 5 indicates differences between various neural networks. A comparison of the values in both tables, reveals that they are not correlated. In Table 5, the results are better for $MLP\_2\_107\_54$ , whereas in Table 6 and 7 results are better for *DNN*1. The assessment of neural networks applied in robot control should not only focus on results from the training process but also be validated with the use of real robots or in simulations .

### Neural network response latency

To analyze the usefulness of a given neural network in real-life conditions, it is necessary to determine how long the network will generate control signals after receiving input data which results in the response delay of the UAV’s control and could make the system unstable. Even the most accurate control will be completely useless if it is given after an acceptable time. Therefore, the time after each type’s best neural network generates a solution was determined. Times were determined with the use of a computer containing an Intel Core i7-9750H, 2.6 GHz processor with all other applications turned off. To minimize the impact of system processes on the calculation results, the calculations were conducted 10 times while processing 1, 000 different patterns. The tables presented below (Table 8) include the means and standard deviations of these results compared with the average values of $RMSE_D$ and $r_{m}$ from Tables 6 and 7 .

**Table 8:** Average calculation time for each type of network compared to avg. $RMSE_D$ and avg. $r_{m}$.

Network	Time [ms]		Avg. $RMSE_D$	Avg. $r_{m}$
	average	SD		
MLP_1_131	0.0244	0.0034	1.2344	0.9489
MLP_2_107_54	4.1257	0.0785	1.4539	0.9181
DNN1	1.3068	0.0170	1.0960	0.9624
DNN2	1.3217	0.0494	1.0830	0.9619
ResNet	5.1659	0.0141	6.2470	0.1480

The results show that the fastest network is the $MLP\_1\_131$ network with a single hidden layer. It runs at least 50 times faster than the other networks do. Moreover, the accuracy of the $MLP\_1\_131$ network is only slightly worse for DNNs. Considering that the average $RMSE_D$ equals 1.2344 [m] vs 1.0830 [m] for *DNN*2 and that the average $r_m$ equals 0.9489 vs 0.9624 for *DNN*1 (8), the $RMSE_D$ is only $13.97\%$ greater, and the $r_m$ is only $1.4\%$ smaller (8). This is an acceptable cost of significantly lower latency. Undoubtedly, this is a result of its compactness and the relatively small number of calculations that need to be performed. A certain surprise may be the second place of deep networks, rather than MLP networks with 2 hidden layers. Here the difference is due mainly to the activation functions used and the low computational complexity of determining the output value in the case of ReLU . By far the most time is needed to determine the output in the case of ResNet . This time was influenced by the need to perform additional operations in each residual unit. When the results obtained, were analyzed, the fundamental sample time was set to 0.065 s (65 ms) . Therefore, all the given times are low enough to be able to determine from 12 ( ResNet ) to more than 2, 660 ( $MLP\_1\_N$ with single hidden layer) times the drone control signals apply the corresponding neural networks. However, if the networks were used in a physical system, the additional time involved in obtaining input values from the sensors would have to be considered, as would the computing power of the CPU lower than that used in this study. This leads to the conclusion that the choice of the network should be made between a deep network and an $MLP\_1\_N$ network with one hidden layer.

Onboard computers or autopilot units used in modern UAVs are typically based on ARM microcontrollers and single-board computers as companion computers. Because both support code in C, and C++, which can be easily used to implement neural networks, there is no need to use dedicated hardware such as neural processing units. The neural networks in this research are relatively small, but in the case of their larger structures, boards of NPU processors should accelerate computing and they are available, e.g., for Raspberry Pi5; thus, the neural network can be coded in Python. In this research, it is possible to control the Parrot UAV with a neural network running in MATLAB through Wi-Fi; therefore, the communication latency is more crucial than the computing latency.

## Conclusions

This study proposes the use of a direct NN-based controller for position and trajectory tracking algorithms in holonomic UAVs. We designed three different types of neural networks to evaluate their position and trajectory control performance. The evaluation was conducted with the use of four distinct flight scenarios, which are specifically designed to encompass challenging maneuvers that are particularly difficult to manage reliably with neural network-based solutions.

The four different flight scenarios included rectangular, triangular, circular, and figure-eight trajectories, and were performed at constant and/or variable altitudes. For each scenario, training data were prepared for a specified set of different velocities of the tracked point. After training , the neural networks were evaluated to demonstrate their generalization performance by testing them on flight scenarios that were not included during the training phase.

Initially, regression-based multilayer perceptron (MLP) networks were tested. In this stage, we evaluated both one-layer and two-layer networks. As a result, eight one-layer networks and a series of two-layer neural networks were trained, demonstrating varying levels of performance. An analysis of the one-layer architecture, clearly reveals that increasing the number of neurons improves tracking quality and simultaneously reduces the error. However, some MLP solutions exhibit unexpected behaviors, such as flying in a loop-like pattern, deviating significantly from the desired path, and encountering serious difficulties when navigating sharp corners. The single-layer MLP neural network with 277 neurons achieved the best statistical indicators, with a validation quality of 0.996589 and a validation error of 0.000321. However, this network did not demonstrate satisfactory performance during the flight scenario. For the double-layer MLP neural networks, the best performance was observed in the model with 107 neurons in the first layer and 54 in the second. This network achieved a validation quality of 0.9972, closely matching the performance of $MLP\_2\_105\_54$, while demonstrating a significantly lower validation error of 0.02091, indicating improved accuracy.

Deep neural networks constitute the second type of architecture considered in the adaptation of NNs as controllers. These networks utilized a complex structure consisting of dense layers with a combination of ReLU and sigmoid activation functions. As shown in the corresponding figures, the deep neural networks demonstrated noteworthy performance, exhibiting smooth and gentle transitions during rapid directional changes, particularly when navigating the corners of polygon-based trajectories. The DNN, which is named 1600*x*..*x*100(0.1), *B*64 achieves the best statistical values, with a validation quality of 0.9941 and a validation error of 0.02421.

In the final step, the time requirements for NN operation are evaluated and measured in the context of real-time applications. The conducted simulations confirmed that the time needed to compute the output of the NN is sufficiently small to meet the strict time constraints required for high-frequency control loops. This applies to all the considered neural networks, with MLPs demonstrating the shortest computation times, whereas residual networks require the longest computation times .

The research presented in this work demonstrates that neural networks face significant challenges when acting as trajectory-tracking controllers. These difficulties are particularly evident in nonlinear segments of the trajectory, during repetitive maneuvers, and at the starting and finishing points. This behavior is likely attributable to the limited generalization ability of neural networks. Consequently, when the NN-based controller encounters highly atypical situations that were not represented during training , it struggles to compute the correct output. In most cases, this leads to an inability to accurately track the desired trajectory and may result in strange or unexpected behaviors.

These issues become even more pronounced when there is a significant discrepancy between the desired and actual positions of the holonomic UAV. If the UAV deviates considerably from its expected trajectory or overshoots the intended set point, the neural network often struggles to adapt effectively, leading to instability or prolonged correction times . This limitation arises from the NN’s dependence on the distribution of training data-if certain flight conditions or deviations are underrepresented during training, the network may fail to generalize properly in real-world applications. Consequently, ensuring the presence of a wide variety of scenarios in the training dataset is crucial for enhancing the NN’s adaptability and mitigating performance degradation.

Despite these challenges, the research presented in this work demonstrates that it is possible to develop a specific class of neural networks capable of achieving highly satisfactory performance in UAV control . By conducting extensive training on a vast and diverse dataset that accurately represents real-world flight conditions, NN-based controllers can overcome many inherent limitations, achieving precise trajectory tracking and adaptive behavior . This approach highlights the critical role of selecting appropriate NN architectures that balance complexity and computational efficiency while ensuring that the training process captures a broad spectrum of flight conditions, including disturbances and nonlinear dynamics. Furthermore, leveraging techniques such as reinforcement training, domain randomization, or transfer training can increase the robustness of NN-based solutions, allowing them to be generalized better across different environments and flight tasks. Ultimately, this research underscores the potential of NN-driven control strategies in UAV applications, provided that their limitations are carefully addressed through rigorous training, architecture optimization, and, where necessary, hybridized control methodologies. Notably, the best overall performance was achieved with the neural network *DNN*2, which achieved an average $RMSE_D$ of 1.083[*m*] and an average $r_m$ of 0.9619 .

Despite the achieved results, it is important to highlight that such solutions require further development and enhancement with additional safety mechanisms, similar to the approaches employed in autonomous vehicles. The inherently unpredictable nature of neural networks makes it inadvisable to rely solely on them for flight control. To ensure operational safety and robustness, NN-based controllers should be complemented with fail-safe algorithms or hybrid control systems capable of intervening during critical situations or unexpected behaviors. To improve the generalisability of neural networks, the focus should be both on the search for other network structures that allow better results than those presented in this article, and on the search for an optimal network structure. Among the methods tentatively considered are: WASD Neuronet Models, Neuroevolutionary Algorithms, and reinforcement learning.

Future research in this field should prioritize more extensive and detailed studies on the reliability and safety of such solutions. This includes exploring methods to improve the generalization capabilities of neural networks, designing frameworks for integrating NNs with traditional control strategies, and developing real-time monitoring systems to detect and mitigate potential failures. Several key aspects are further explored to enhance the applicability of neural networks in quadcopter flight control. One important direction is improving the robustness of NN-based controllers against external disturbances, such as wind, sensor noise, or hardware malfunctions. Another crucial area is the development of adaptive and online training mechanisms, allowing the neural network to dynamically adjust to changing flight conditions without requiring retraining. Additionally, enhancing the interpretability of neural networks would make their decision-making process more transparent, facilitating debugging, validation, and certification for safety-critical applications. Further work should also focus on optimizing hybrid control approaches by refining methods for integrating neural networks with classic control laws to ensure stability, reliability, and improved performance. Another key challenge is optimizing NN architectures for deployment on embedded systems with limited computational resources, ensuring real-time operation without excessive power consumption. Expanding NN-based control to multiagent and swarm coordination scenarios could enable more efficient drone collaboration and task execution. Addressing these aspects is essential for making NN-based controllers viable and trustworthy components in flight control applications.

## Data Availability

Data will be made available on request. Correspondence and requests for materials should be addressed to C. Kownacki.

## References

[1] Afifi, G. & Gadallah, Y. Cellular network-supported machine learning techniques for autonomous UAV trajectory planning. *IEEE Access* **10**, 131996–132011. 10.1109/ACCESS.2022.3229171 (2022).

[2] Catena, A., Melita, C. & Muscato, G. Automatic tuning architecture for the navigation control loops of unmanned aerial vehicles. *J. Intell. Robot. Syst.*10.1007/s10846-013-9919-2 (2014).

[3] Sarkar, N. & Gul, S. Artificial intelligence-based autonomous UAV networks: A survey. *Drones* **7**, 322. 10.3390/drones7050322 (2023).

[4] Rahman, M. M., Siddique, S., Kamal, M., Rifat, R. H. & Gupta, K. D. UAV (unmanned aerial vehicle): Diverse applications of UAV datasets in segmentation, classification, detection, and tracking. *Algorithms*10.3390/a17120594 (2024).

[5] Liu, Z. & Li, J. Application of unmanned aerial vehicles in precision agriculture. *Agriculture*10.3390/agriculture13071375 (2023).

[6] Zhang, J. et al. Lifetime extension approach based on the Levenberg–Marquardt neural network and power routing of DC–DC converters. *IEEE Trans. Power Electron.* **38**, 10280–10291. 10.1109/TPEL.2023.3275791 (2023).

[7] Sabeeh Hasan Allak, A., Yi, J., Al-Sabbagh, H. M. & Chen, L. Siamese neural networks in unmanned aerial vehicle target tracking process. *IEEE Access* **13**, 24309–24322. 10.1109/ACCESS.2025.3536461 (2025).

[8] Basil, N. & Marhoon, H. M. Towards evaluation of the PID criteria based UAVs observation and tracking head within resizable selection by COA algorithm. *Results Control Optim.* **12**, 100279. 10.1016/j.rico.2023.100279 (2023).

[9] Jia, K., Lin, S., Du, Y., Zou, C. & Lu, M. Research on route tracking controller of quadrotor UAV based on fuzzy logic and RBF neural network. *IEEE Access* **11**, 111433–111447. 10.1109/ACCESS.2023.3322944 (2023).

[10] Yang, L., Wu, L., Lv, Y. & Zhang, Z. Combined MPC and dynamic neural network-based UAVs trajectory tracking control. *IEEE Access* **11**, 145763–145771. 10.1109/ACCESS.2023.3343770 (2023).

[11] Nodland, D., Zargarzadeh, H. & Jagannathan, S. Neural network-based optimal adaptive output feedback control of a helicopter UAV. *IEEE Trans. Neural Netw. Learn. Syst.* **24**, 1061–1073. 10.1109/TNNLS.2013.2251747 (2013). 24808521 10.1109/TNNLS.2013.2251747

[12] Xu, T., Wu, D., Meng, W., Ni, W. & Zhang, Z. Energy-optimal trajectory planning for near-space solar-powered UAV based on hierarchical reinforcement learning. *IEEE Access* **12**, 21420–21436. 10.1109/ACCESS.2024.3359901 (2024).

[13] Zhou, Y., Tian, Z. & Lin, H. UAV based adaptive trajectory tracking control with input saturation and unknown time-varying disturbances. *IET Intell. Transp. Syst.* **17**, 780–793. 10.1049/itr2.12303 (2023).

[14] Gao, Y., Gan, Z., Chen, M., Ma, H. & Mao, X. Hybrid dual-scale neural network model for tracking complex maneuvering UAVs. *Drones*10.3390/drones8010003 (2024).

[15] Patel, S., Sarabakha, A., Kircali, D. & Kayacan, E. An intelligent hybrid artificial neural network-based approach for control of aerial robots. *J. Intell. Robot. Syst.* **97**, 1–12. 10.1007/s10846-019-01031-z (2020).

[16] Fang, P. et al. A high-performance neural network vehicle dynamics model for trajectory tracking control. *Proc. Inst. Mech. Eng. Part D J. Automob. Eng.* **237**, 095440702210956. 10.1177/09544070221095660 (2022).

[17] Chai, S. & Lau, V. K. N. Multi-UAV trajectory and power optimization for cached UAV wireless networks with energy and content recharging-demand driven deep learning approach. *IEEE J. Sel. Areas Commun.* **39**, 3208–3224. 10.1109/JSAC.2021.3088694 (2021).

[18] Khachumov, M. An approach to formation control of UAVs based on applying adapted kohonen neural network. In *2023 IEEE Ural-Siberian Conference on Biomedical Engineering, Radioelectronics and Information Technology (USBEREIT)*, 258–261, 10.1109/USBEREIT58508.2023.10158836 (2023).

[19] Yu, G., Reis, J. & Silvestre, C. Quadrotor neural network adaptive control: Design and experimental validation. *IEEE Robot. Autom. Lett.* **8**, 2574–2581. 10.1109/LRA.2023.3254450 (2023).

[20] Ouyang, Y., Xue, L., Dong, L. & Sun, C. Neural network-based finite-time distributed formation-containment control of two-layer quadrotor UAVs. *IEEE Trans. Syst. Man Cybern. Syst.* **52**, 4836–4848. 10.1109/TSMC.2021.3103013 (2022).

[21] Cheng, Z., Pei, H. & Li, S. Neural-networks control for hover to high-speed-level-flight transition of ducted fan UAV with provable stability. *IEEE Access* **8**, 100135–100151. 10.1109/ACCESS.2020.2997877 (2020).

[22] Dong, F., Li, X., You, K. & Song, S. Standoff tracking using DNN-based MPC with implementation on FPGA. *IEEE Trans. Control Syst. Technol.* **31**, 1998–2010. 10.1109/TCST.2023.3279115 (2023).

[23] Wang, G.-B. Adaptive sliding mode robust control based on multi-dimensional Taylor network for trajectory tracking of quadrotor UAV. *IET Control Theory Appl.* **14**, 1855–1866. 10.1049/iet-cta.2019.1058 (2020).

[24] Bouaiss, O., Mechgoug, R., Taleb-Ahmed, A. & Brikel, A. E. Adaptive neural network based compensation control of quadrotor for robust trajectory tracking. *Int. J. Adapt. Control Signal Process.* **37**, 2772–2793. 10.1002/acs.3659 (2023).

[25] Jiang, F., Pourpanah, F. & Hao, Q. Design, implementation, and evaluation of a neural-network-based quadcopter UAV system. *IEEE Trans. Industr. Electron.* **67**, 2076–2085. 10.1109/TIE.2019.2905808 (2020).

[26] Chen, Z., Kang, N. & Li, L. Research on adaptive trajectory tracking algorithm for a quadrotor based on backstepping and the sigma-pi neural network. *Int. J. Aerosp. Eng.* **1–9**, 2019. 10.1155/2019/1510341 (2019).

[27] Chang, Y.-T. & Chen, B. Intelligent robust tracking controls for holonomic and nonholonomic mechanical systems using only position measurements. *IEEE Trans. Fuzzy Syst.* **13**, 491–507. 10.1109/TFUZZ.2004.840125 (2005).

[28] Selma, B., Chouraqui, S., Selma, B. & Abouaïssa, H. Design an optimal ANFIS controller using bee colony optimization for trajectory tracking of a quadrotor UAV. *J. Inst. Eng. India. Ser. B* **103**, 1505–1519 (2022).

[29] Mendoza, A. & Yu, W. Fuzzy adaptive control law for trajectory tracking based on a fuzzy adaptive neural PID controller of a multi-rotor unmanned aerial vehicle. *Int. J. Control Autom. Syst.* **21**, 658–670. 10.1007/s12555-021-0299-2 (2023).

[30] Celen, B. & Oniz, Y. Trajectory tracking of a quadcopter using fuzzy logic and neural network controllers. In *2018 6th International Conference on Control Engineering & Information Technology (CEIT)*, 1–6, 10.1109/CEIT.2018.8751810 (2018).

[31] Ma, C., Lam, J. & Lewis, F. L. Trajectory regulating model reference adaptive controller for robotic systems. *IEEE Trans. Control Syst. Technol.* **27**, 2749–2756. 10.1109/TCST.2018.2858203 (2019).

[32] Sinha, P., Krim, H. & Guvenc, I. Neural network based tracking of maneuvering unmanned aerial vehicles. 380–386, 10.1109/IEEECONF56349.2022.10052072 (2022).

[33] Hasan, A. *et al.**Fractional Order Extended State Observer Enhances the Performance of Controlled Tri-copter UAV Based on Active Disturbance Rejection Control*, 439–487 (2023).

[34] Lv, J. & Tu, L. Deep learning-based visual navigation control method for autonomous trajectory of UAVs. *Appl. Math. Nonlinear Sci.*10.2478/amns.2023.2.01249 (2024).

[35] Saif, E. & Eminoglu, I. Modelling of quad-rotor dynamics and hardware-in-the-loop simulation. *J. Eng.* **937–950**, 2022. 10.1049/tje2.12152 (2022).

[36] Novotnák, J., Szoke, Z., Kašper, P. & Šmelko, M. Quadcopter modeling using a system for UAV parameters measurement. *Drones*10.3390/drones8070280 (2024).

[37] Tahir, Z. *et al.* State space system modeling of a quad copter UAV. *Indian J. Sci. Technol.* **9**, 10.48550/arXiv.1908.07401 (2016).

[38] Cezary, K. Artificial potential field based trajectory tracking for quadcopter UAV moving targets. *Sensors* **24**, 1343. 10.3390/s24041343 (2024). 38400501 10.3390/s24041343 PMC10893262

[39] Swietlicka, I., Kuniszyk-Józkowiak, W. & Swietlicki, M. Artificial neural networks combined with the principal component analysis for non-fluent speech recognition. *Sensors*10.3390/s22010321 (2022). 35009863 10.3390/s22010321 PMC8749906

[40] Gebisa, A., Gebresenbet, G., Gopal, R. & Nallamothu, R. B. A neural network and principal component analysis approach to develop a real-time driving cycle in an urban environment: The case of Addis Ababa, Ethiopia. *Sustainability*10.3390/su142113772 (2022).

[41] Nguyen, V. Bayesian optimization for accelerating hyper-parameter tuning. In *2019 IEEE Second International Conference on Artificial Intelligence and Knowledge Engineering (AIKE)*, 302–305 (IEEE, 2019).

[42] Elsken, T., Metzen, J. H. & Hutter, F. Neural architecture search: A survey. *J. Mach. Learn. Res.* **20**, 1–21 (2019).

[43] Baker, B., Gupta, O., Naik, N. & Raskar, R. Designing neural network architectures using reinforcement learning. arXiv preprint arXiv:1611.02167 (2016).

[44] Young, S. R., Rose, D. C., Karnowski, T. P., Lim, S.-H. & Patton, R. M. Optimizing deep learning hyper-parameters through an evolutionary algorithm. In *Proceedings of the Workshop on Machine Learning in High-Performance Computing Environments*, 1–5 (2015).

[45] Tirumala, S. S. Implementation of evolutionary algorithms for deep architectures (CEUR Workshop Proceedings, 2014).

[46] Zhang, Y., Chen, D. & Ye, C. *Toward Deep Neural Networks: WASD Neuronet Models, Algorithms, and Applications* (Chapman and Hall/CRC, 2019).

[47] Kingma, D. P. Adam: A method for stochastic optimization. arXiv preprint arXiv:1412.698010.48550/arXiv.1412.6980 (2014).

[48] Ziad, H., Al-dujaili, A. & Humaidi, A. J. Electrical faults classification in permanent magnet synchronous motor using resnet neural network. *Int. Rev. Appl. Sci. Eng.* **15**, 355–364 (2024).

[49] Chen, D., Hu, F., Nian, G. & Yang, T. Deep residual learning for nonlinear regression. *Entropy* **22**, 193 (2020). 33285968 10.3390/e22020193 PMC7516619

[50] He, K., Zhang, X., Ren, S. & Sun, J. Identity mappings in deep residual networks. In *Computer Vision–ECCV 2016: 14th European Conference, Amsterdam, The Netherlands, October 11–14, 2016, Proceedings, Part IV 14*, 630–645 (Springer, 2016).

